# Targeting RANKL-independent osteoclastogenesis overcomes denosumab resistance in models of ER^+^ breast cancer bone metastasis

**DOI:** 10.1172/JCI199285

**Published:** 2026-05-15

**Authors:** Qun Lin, Jinpeng Luo, Zhuxi Duan, Jieer Luo, Wei Zhang, Yuan Xia, Yinduo Zeng, Xiaolin Fang, Jiahui Liang, Jiayi Chen, Qianchong Lin, Yilin Quan, Ruiyu Hu, Hongcai Liu, Qiang Liu, Jun Li, Chang Gong

**Affiliations:** 1 Guangdong Provincial Key Laboratory of Malignant Tumor Epigenetics and Gene Regulation, Guangdong-Hong Kong Joint Laboratory for RNA Medicine, Sun Yat-Sen Memorial Hospital, Sun Yat-Sen University, Guangzhou, China.; 2 Breast Tumor Center, Sun Yat-Sen Memorial Hospital, Sun Yat-Sen University, Guangzhou, China.; 3 Department of Biochemistry, Zhongshan School of Medicine, Guangzhou, China.

**Keywords:** Cell biology, Oncology, Breast cancer, Monocytes, Osteoclast/osteoblast biology

## Abstract

Bone metastasis remains a major cause of morbidity in estrogen receptor–positive breast cancer, with RANKL inhibitor resistance emerging as a critical clinical challenge. Nearly 40% of patients develop progressive skeletal lesions despite denosumab therapy, highlighting an urgent need to identify resistance mechanisms and alternative therapeutic strategies. We identified a RANKL-independent osteoclast activation pathway mediated by the CRKL/circCCDC50/NFATc1 axis. Mechanistically, CRKL promoted EIF4A3-dependent circCCDC50 biogenesis, which was packaged into large oncosomes and transferred to osteoclast precursors. Nuclear circCCDC50 recruited CARM1 to epigenetically activate NFATc1 transcription, establishing a self-reinforcing loop that sustained osteolysis despite RANKL blockade. Pharmacological inhibition of CARM1 (TP-064) effectively suppressed osteoclastogenesis and bone metastasis in denosumab-resistant models. These findings revealed a targetable resistance mechanism and provided a clinically actionable strategy to overcome microenvironment-driven metastasis through dual targeting of tumor and bone niches.

## Abstract 2

<strong>Targeting RANKL-independent osteoclastogenesis overcomes Denosumab resistance in models of ER+ breast cancer bone metastasis</strong>

## Graphical abstract

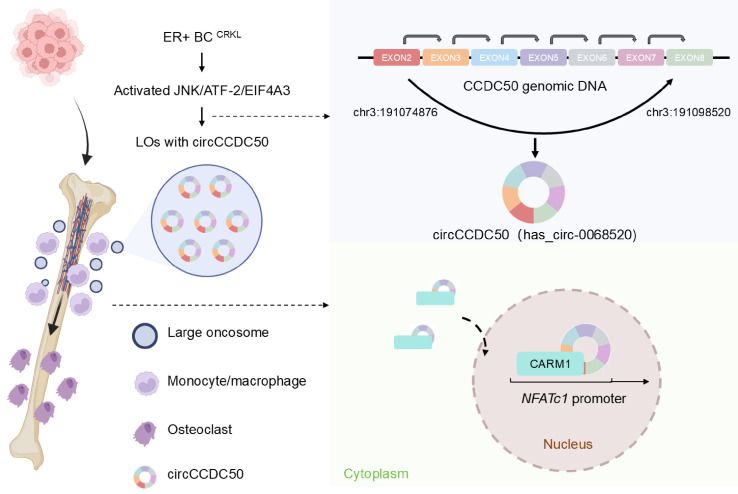

## Introduction

Breast cancer (BC) remains the most frequently diagnosed malignancy and the leading cause of cancer-related mortality among women worldwide. Estrogen receptor–positive (ER+) BC exhibits a pronounced propensity for bone metastasis, with approximately 65%–70% of patients with ER+ metastasis developing skeletal involvement (1, 2). These metastases typically present as multifocal lesions, with approximately 30% of cases exhibiting bone-only disease, while others manifest concurrent visceral metastases, a clinical scenario that substantially complicates therapeutic management (3). The onset of bone metastasis varies remarkably, ranging from several years to decades after primary diagnosis, underscoring the persistent long-term risk even following successful initial treatment (1, 4).

Bone metastases severely compromise both patient quality of life and survival, primarily due to skeletal-related events (SREs) such as pathologic fractures, spinal cord compression, and hypercalcemia (5, 6). These complications result from dysregulated osteoclast activation, which disrupts normal bone remodeling and establishes a self-perpetuating cycle of tumor growth and bone destruction (7). Current clinical guidelines recommend early and continuous administration of bone-modifying agents, particularly RANKL inhibitors like denosumab, to mitigate SREs by reducing osteoclast activity (8, 9). However, nearly 40% of patients treated with RANKL inhibitors demonstrate poor therapeutic response, continuing to experience SREs within the first year of treatment (10–13).

Emerging evidence indicates that resistance to RANKL inhibition arises through alternative osteoclast-activating pathways that bypass RANKL dependency (14, 15). ER+ BC employs multiple compensatory mechanisms, including cytokine-mediated osteoclast stimulation (e.g., via IL-8 or TGF-β) (16, 17), tumor-derived extracellular vesicles containing proosteolytic miRNAs (18, 19), and direct secretion of proteolytic enzymes (e.g., MMP-13 and cathepsin K) (20, 21). These RANKL-independent pathways drive persistent osteolysis and contribute to therapeutic failure (15, 22), yet no clinically available therapies currently target these resistance mechanisms (14, 23).

These unmet clinical needs underscore the urgent requirement to elucidate the molecular mechanisms underlying both bone tropism and RANKL inhibitor resistance in ER+ BC, particularly those involving RANKL-independent pathways. Developing strategies for early prediction and targeted intervention could transform the clinical management of ER+ BC bone metastases, shifting from reactive treatment to proactive prevention of skeletal complications (23, 24).

In this study, we identify a CRKL/circCCDC50/NFATc1 signaling axis that drives osteoclast activation and bone metastasis progression independently of RANKL. We demonstrate that ER+ BC cells upregulate CRKL to promote EIF4A3-mediated biogenesis of circCCDC50, which is selectively packaged into large oncosomes (LOs) and transported to bone marrow–derived macrophages. Within these recipient cells, circCCDC50 recruits CARM1 to the *NFATc1* promoter region, epigenetically enhancing NFATc1-driven osteoclastogenesis. Importantly, pharmacological inhibition of CARM1 with TP-064 effectively disrupts this cascade and suppresses bone metastasis progression. These findings elucidate a mechanism underlying RANKL inhibitor resistance in bone metastasis while simultaneously identifying a druggable signaling axis that may transform clinical management for patients with ER+ BC with aggressive skeletal involvement.

## Results

### Osteoclast activation is enhanced in ER+ BC bone metastasis models and persists in denosumab-resistant cases.

**Figure 1:**
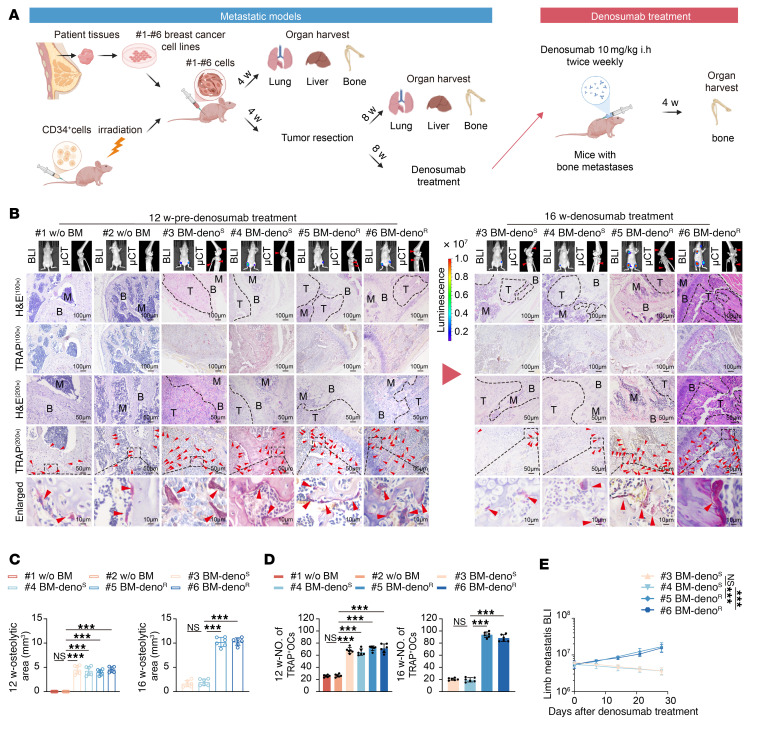
Osteoclasts exhibit sustained activation during the pathogenesis and therapeutic intervention of denosumab-resistant bone metastasis in ER+ breast cancer in vivo. (**A**) Left: Schematic diagram illustrating the establishment of CDX models. Cell lines established from primary tumor cells collected from clinical patients who were treatment-naive were surgically implanted into humanized NCG hairless mice. Subcutaneous tumors were excised 4 weeks after implantation, with concurrent collection of bone, liver, and lung tissues for histological analysis. At 8 weeks after excision, bone, liver, and lung tissues were harvested to evaluate metastatic progression, and mice in the bone metastasis cohort were administered denosumab therapy. Right: Schematic representation of denosumab treatment and efficacy evaluation in bone metastasis–bearing mice. Following a 4-week therapeutic regimen, bone tissues were systematically collected for histopathological analysis to assess treatment efficacy. (**B**) BLI images of mice, μCT images of trabecular bone structure in the knee joint, H&E staining of murine bone tissue, TRAP staining. Scale bars: 100 μm (100×), 50 μm (200×), and 10 μm (Enlarged). (**C**–**E**) Quantification of bone parameters, including μCT-based osteolytic lesion area (**C**) and TRAP+ osteoclast numbers (**D**) in representative mice (*n* = 6 per group), and normalized BLI signals of bone metastases (**E**). Each error bar represents the mean ± SD of 6 independent experiments. Significant differences were determined by 1-way ANOVA with Tukey’s multiple comparison test. *P < 0.05, **P < 0.01, ***P < 0.001.

**Figure 2:**
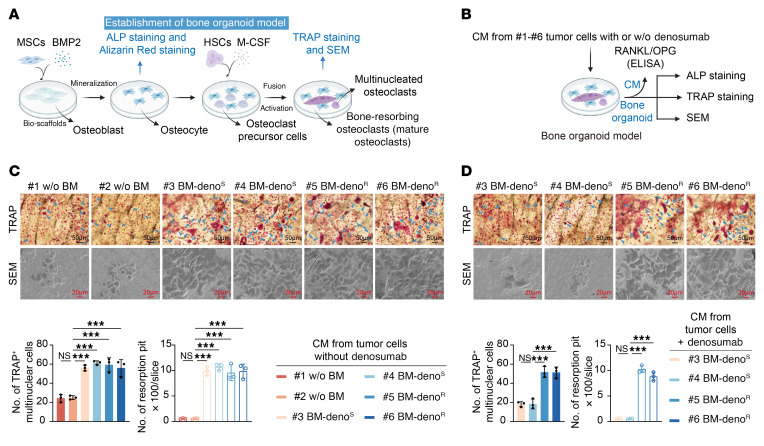
Denosumab-resistant ER+ breast cancer secretes LOs to promote RANKL-independent osteoclast activation in vitro. (**A**) Experimental workflow for establishing skeletal organoid models. (**B**) Schematic diagram depicting analytical methods for skeletal organoid models, including ELISA, ALP staining, TRAP staining, and scanning electron microscopy (SEM). (**C**) Representative TRAP-stained images (scale bar: 50 μm) and corresponding SEM micrographs (scale bar: 20 μm) of skeletal organoid models treated with specified conditioned media. Quantitative analysis of TRAP+ multinucleated cell numbers and resorption pit counts per section in skeletal organoid models. (**D**) Representative TRAP-stained images (scale bar: 50 μm) and SEM micrographs (scale bar: 20 μm) of skeletal organoid models treated with conditioned media and denosumab. Quantification of TRAP+ multinucleated cells and resorption pits per section in treated skeletal organoid models. ach error bar represents the mean ± SD of 3 independent experiments. Significant differences were determined by 1-way ANOVA with Tukey’s multiple comparison test. *P < 0.05, **P < 0.01, ***P < 0.001.

To investigate bone microenvironmental changes associated with differential responses to RANKL inhibition, we established cell line–derived xenograft (CDX) models using primary tumor cells isolated from patient-derived BC tissues (#1 and #2: non-metastatic; #3 and #4: bone-metastatic and denosumab-sensitive; #5 and #6: bone-metastatic and denosumab-resistant) (Figure 1A). Primary tumor cells were orthotopically injected into the mammary fat pad of humanized NCG hairless mice to establish a CDX model. Four weeks after orthotopic mammary tumor cell inoculation, a subset of mice underwent comprehensive assessment of bone microenvironmental alterations, while the remaining cohort received surgical resection of primary tumors. Microcomputed tomography (μCT) analysis revealed significant bone microarchitectural alterations in bone-metastatic models (#3–#6) compared to nonmetastatic controls (#1, #2) during the premetastatic phase, including elevated trabecular separation and trabecular bone pattern factor, along with reduced bone volume fraction and trabecular number and thickness. Histological examination confirmed these observations, demonstrating markedly increased tartrate-resistant acid phosphatase–positive (TRAP+) osteoclasts but comparable alkaline phosphatase–positive (ALP+) osteoblast numbers and CD31+ vascular density across all groups (Supplemental Figure 1, A–F; supplemental material available online with this article; https://doi.org/10.1172/JCI199285DS1). Eight weeks after surgical resection of primary tumors, in vivo imaging analysis revealed distinct bioluminescent signals in the limbs of bone-metastatic models (#3–#6), whereas no signals were detected in nonmetastatic controls (#1, #2). Furthermore, μCT quantification of a subset cohort demonstrated significantly expanded osteolytic areas in bone-metastatic models (#3–#6) versus nonmetastatic controls (#1, #2). Histopathological examination confirmed elevated quantities of TRAP+ osteoclasts within the bone microenvironment of metastatic groups compared with controls. Subsequently, bone-metastatic cohorts (#3–#6) received denosumab treatment. While denosumab treatment effectively suppressed these premetastatic osteolytic changes in sensitive models (#3, #4), resistant cases (#5, #6) maintained persistently elevated osteoclast activity (Figure 1, B–E). Notably, resistant models exhibited significantly worse survival outcomes compared with sensitive cases at matched time points (Supplemental Figure 1G). Furthermore, we performed other in vivo experiments in which some humanized NCG hairless mice received OPG-Fc treatment for 4 weeks. Although this treatment robustly suppressed premetastatic osteolysis in sensitive models (#3, #4), it failed to normalize osteoclast activity in resistant models (#5, #6) (Supplemental Figure 1, H–J). These results collectively demonstrate that osteoclast activation in the bone microenvironment plays a crucial role in mediating denosumab resistance in BC bone metastasis.Consistent with these findings, our bone organoid studies demonstrated that conditioned media from metastatic groups significantly enhanced osteoclast differentiation and bone resorption compared with nonmetastatic groups, while osteoblast activity remained unchanged (Figure 2, A–C, and Supplemental Figure 2, A–C). Denosumab treatment effectively inhibited osteoclast activity in sensitive models but showed minimal effect in resistant cases (Figure 2D), confirming RANKL-independent osteoclast activation in treatment-resistant bone metastasis. Additionally, we found that the conditioned medium from primary BC cells #5 BM-Deno^R^ and #6 BM-Deno^R^ alone, when administered intraperitoneally to mice, was sufficient to induce damage to the trabecular bone structure (Supplemental Figure 2D).

### Denosumab-resistant ER+ BC secretes large oncosomes to promote RANKL-independent osteoclast activation.

To identify key mediators of osteoclast activation, we systematically analyzed critical osteoclastogenic factors. Cytokine array screening and ELISA quantification of tumor cell–conditioned media and patient plasma samples (including TNFA, IL-6, M-CSF, and RANKL) revealed no consistent differences in cytokine profiles between denosumab-sensitive and resistant groups (Supplemental Figure 3, A–C). Treated with neutralizing antibody targeting the 5 most differentially expressed cytokines detected in vitro, denosumab-resistant breast cancer cells maintained their ability to induce osteoclast differentiation and enhance TRAP+ activity in vitro (Supplemental Figure 3D), suggesting the involvement of alternative, noncytokine mediated pathways in driving osteoclast activation in the resistant phenotype.

**Figure 3:**
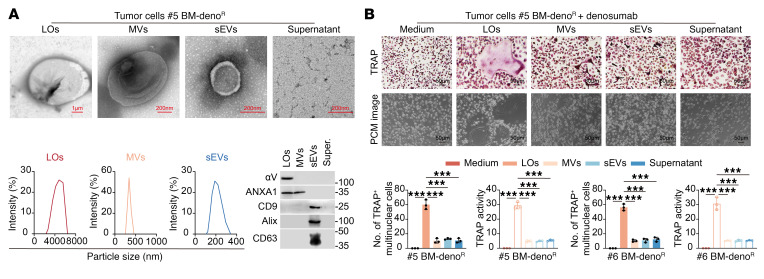
Denosumab-resistant ER+ breast cancer secretes LOs to promote RANKL-independent osteoclast activation in vitro. (**A**) Top: Representative transmission electron microscopy (TEM) images of LOs (scale bar: 1 μm), microvesicles (MVs, scale bar: 100 nm), small extracellular vesicles (SEVs, scale bar: 100 nm), and supernatant-depleted LOs (scale bar: 500 nm). Bottom: Nanoparticle tracking analysis (NTA) of LOs, MVs, and SEVs using membrane filters with pore sizes of 2,000 nm, 800 nm, and 100 nm, respectively. Immunoblot (IB) analysis of αV-integrin, ANXA1, CD9, ALIX, and CD63 expression in LOs, MVs, SEVs, and supernatant fractions. (**B**) Top: TRAP staining and phase-contrast microscopy images of BMMs treated with medium, LOs, MVs, sEVs, or supernatant. Scale bars: 50 μm. Bottom: Quantification of TRAP+ multinucleated cell counts and TRAP enzymatic activity from the experiments above. Each error bar represents the mean ± SD of 3 independent experiments. Significant differences were determined by 1-way ANOVA with Tukey’s multiple comparison test. *P < 0.05, **P < 0.01, ***P < 0.001.

**Figure 4:**
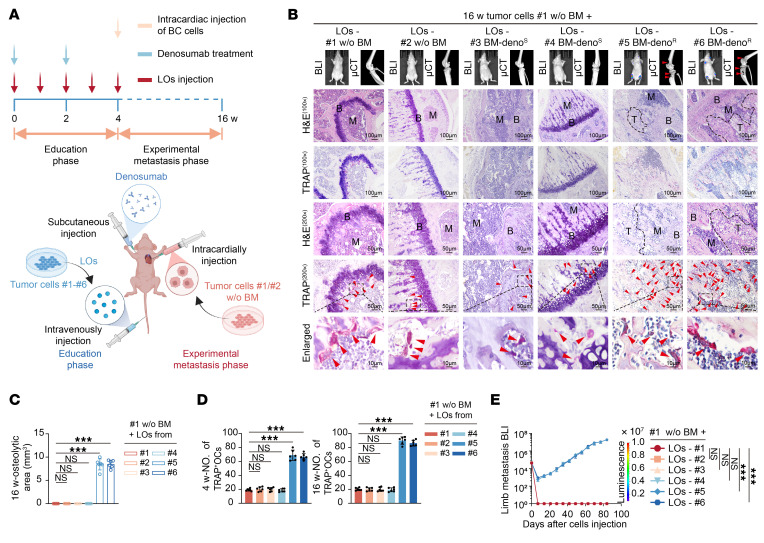
Denosumab-resistant ER+ breast cancer secretes LOs to promote RANKL-independent osteoclast activation in vivo. (**A**) Schematic overview of the premetastatic niche education and experimental metastasis mouse models. (**B**) Bioluminescence imaging (BLI) images of primary BC cells and μCT images of trabecular bone structure in the knee joint, H&E staining of murine bone tissue, tartrate-resistant acid phosphatase (TRAP) staining. Scale bar: 100 μm (100×), 50 μm (200×), and 10 μm (Enlarged). (**C**) Quantification of osteolytic area in BC models educated by LOs from primary tumor cells. (**D**) Quantification of TRAP+ OCs, osteolytic area, and BLI in BC models educated by LOs from primary tumor cells. OCs, osteoclasts. (**E**) BLI in BC models educated by LOs from tumor cells. Each error bar represents the mean ± SD of 6 independent experiments. Significant differences were determined by 1-way ANOVA with Tukey’s multiple comparison test. *P < 0.05, **P < 0.01, ***P < 0.001.

To further identify the underlying key mediators of premetastatic niche formation, we isolated different extracellular vesicle populations (LOs, MVs, sEVs) and supernatant from conditioned media of primary tumor cells using differential centrifugation followed by density gradient purification (Figure 3A). Strikingly, only LOs derived from denosumab-resistant cells significantly enhanced osteoclast differentiation in vitro, as evidenced by increased TRAP+ multinucleated cell formation and elevated TRAP enzymatic activity (Figure 3B). Furthermore, LOs can be taken up by denosumab-resistant, ER+ BC cells (Supplemental Figure 3E). In vivo, mice preconditioned with resistant LOs (#5, #6) developed significant bone microarchitectural deterioration and metastatic lesions despite denosumab treatment, accompanied by increased TRAP+ osteoclast numbers along trabecular surfaces compared with sensitive LO-treated groups (Figure 4, A–E, and Supplemental Figure 3, F–J). These results demonstrate that LOs from resistant tumors uniquely reprogram the bone microenvironment to facilitate metastatic progression independent of RANKL signaling.

### LO-encapsulated CircCCDC50 is essential for persistent osteoclastogenesis in denosumab-resistant ER+ BC bone metastasis models.

**Figure 5:**
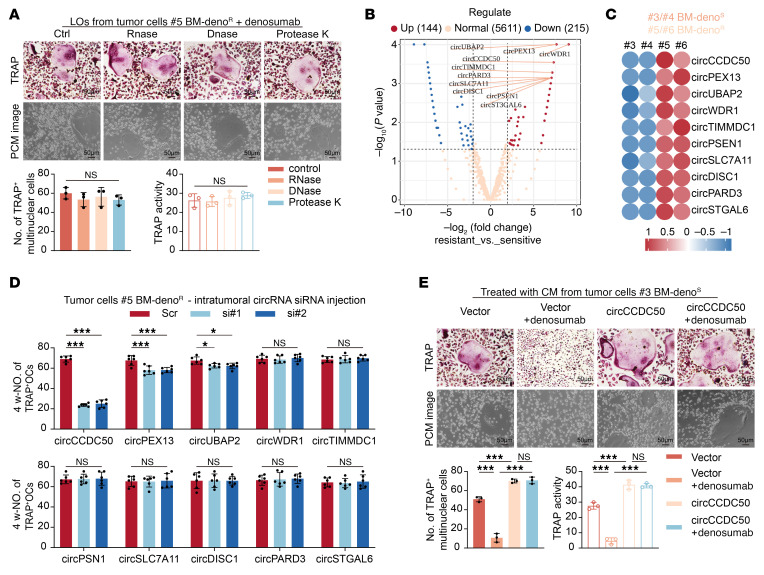
circCCDC50 mediates denosumab-resistant bone metastasis in ER+ breast cancer. (**A**) Top: TRAP-stained and phase-contrast images of osteoclasts treated with LOs derived from #5 primary tumor cells after 5 freeze-thaw cycles or incubation with Proteinase K (20 mg/mL), RNase A (0.02 mg/mL), or DNase I (0.1 mg/mL). Scale bars: 50 μm (TRAP), 20 μm (phase contrast). Bottom: Quantification of TRAP+ multinucleated cells and TRAP activity. (**B**) Top 10 circRNAs with |log_2_FC| > 2.0 and *P* < 0.05 in LOs from #5 primary tumor cells vs. LOs from #3 primary tumor cells. (**C**) qRT-PCR validation of top 10 circRNAs’ expression in #3–#6 primary tumor cells. (**D**) TRAP+ multinucleated osteoclast counts in bone tissues of #5 BC models treated with circRNA-targeting siRNA or non-targeting siRNA control. (**E**) Top: Representative TRAP-stained osteoclasts and phase contrast microscope image of osteoclasts in BMMs treated with conditioned media (CM) from circCCDC50-overexpressing (#3, #4) or control cells, with/without denosumab. Bottom: Quantification of TRAP+ multinucleated cells (top) and TRAP enzymatic activity. Each error bar represents the mean ± SD of 3 independent experiments. Significant differences were determined by 1-way ANOVA with Tukey’s multiple comparison test. *P < 0.05, **P < 0.01, ***P < 0.001.

**Figure 6:**
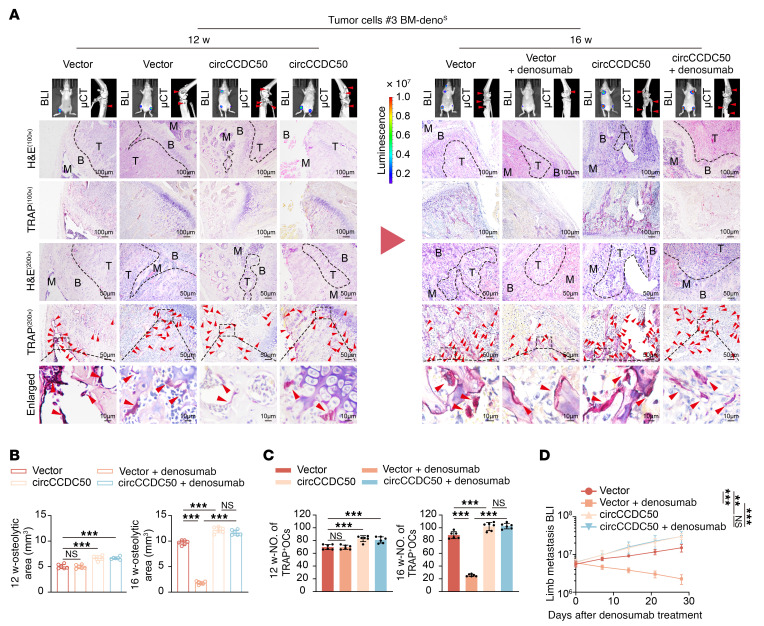
circCCDC50 mediates ER+ breast cancer denosumab resistant bone metastasis in vivo. (**A**) BLI images and μCT scans of bone tissues from BC models, Representative images of H&E staining, TRAP staining. Scale bar: 100 μm (100×), 50 μm (200×), and 10 μm (Enlarged). (**B**) Quantitative analysis of osteolytic lesion area via μCT in mice (*n* = 6 per group). (**C**) Quantitative analysis of TRAP+ multinucleated cell counts per field in mice (*n* = 6 per group). (**D**) Quantification of normalized BLI signals of mice (*n* = 6 per group). Each error bar represents the mean ± SD of 6 independent experiments. Significant differences were determined by 1-way ANOVA with Tukey’s multiple comparison test. *P < 0.05, **P < 0.01, ***P < 0.001.

To characterize the osteoclastogenic components within LOs, we found that treatment with RNase, DNase, or protease failed to attenuate the osteoclast-activating capacity of LOs derived from denosumab-resistant ER+ BC cells (Figure 5A and Supplemental Figure 4A). Given the known stability of circular RNAs against enzymatic degradation, we performed circular RNA sequencing and identified circCCDC50 as specifically enriched in LOs from resistant tumors (Figure 5, B–D, and Supplemental Figure 4, B–G). Notably, CM derived from circCCDC50-knockdown BM-Deno^R^ breast cancer cells (#5, #6) markedly diminished both the number of TRAP^+^ multinucleated osteoclasts and TRAP enzymatic activity of osteoclasts (Supplemental Figure 5A). Following orthotopic implantation of BM-Deno^R^ breast cancer cells (#5, #6) with or without circCCDC50 knockdown, primary tumors were surgically resected at 4 weeks. Primary tumors were resected at 4 weeks after implantation. In vivo BLI at the 8-week postresection time point confirmed metastatic dissemination to the limbs in all cohorts. Quantitative μCT analysis revealed significantly attenuated osteolytic lesion volumes in the circCCDC50-knockdown group versus controls (Supplemental Figure 5B). Histomorphometric assessment further demonstrated reduced TRAP^+^ osteoclast density within the bone microenvironment of circCCDC50-deficient cohorts (Supplemental Figure 5C). Prominently, in the presence of denosumab, CM derived from circCCDC50-overexpressing BM-Deno^S^ BC cells (#3, #4) significantly increased the number of TRAP+-multinuclear osteoclasts and TRAP enzymatic activity (Figure 5E and Supplemental Figure 5D). Subsequently, we established orthotopic mammary tumors using BM-Deno^S^ BC cells (#3, #4) transfected with either circCCDC50-knockdown or nontargeting control constructs. In vivo BLI at 8 weeks after resection revealed diffuse metastatic signals throughout the limbs of all mice. Quantitative μCT analysis demonstrated significantly expanded osteolytic lesions in the circCCDC50-overexpressing cohort compared with controls. Histomorphometric examination further confirmed elevated TRAP^+^ osteoclast density within the bone microenvironment of circCCDC50-overexpressing mice (Figure 6, A–D, and Supplemental Figure 5, E–G). In denosumab-resistant ER+ BC cells, circCCDC50 overexpression did not significantly affect proliferation, as determined by colony formation assays (Supplemental Figure 5H). Similarly, no significant impact on migration or invasion was observed in transwell assays (Supplemental Figure 5, I and J). In patients with ER+ BC, plasma levels of circCCDC50 were significantly higher in those with denosumab-resistant bone metastases than in those with denosumab-sensitive bone metastases or nonmetastatic disease (Supplemental Figure 5K). Moreover, elevated plasma circCCDC50 was inversely correlated with bone metastasis-free survival (BMFS) (Supplemental Figure 5L). Following circCCDC50 knockout, treatment of bone organoids with LOs from resistant cells resulted in a significant reduction in osteoclast formation, as evidenced by TRAP staining. Consistently, SEM analysis showed a marked decrease in bone resorption pits (Supplemental Figure 6, A and B). Treatment of bone organoids with in vitro–transcribed circCCDC50 alone led to a significant increase in both osteoclast formation and bone resorption pits (Supplemental Figure 6, A and B). Following circCCDC50 knockout, treatment of bone organoids with LOs from resistant cells resulted in a significant reduction in osteoclast formation, as evidenced by TRAP staining. Consistently, SEM analysis showed a marked decrease in bone resorption pits (Supplemental Figure 6, A and B).

Mass spectrometry and coIP analyses demonstrated that circCCDC50 is selectively packaged into LOs through direct binding with cytokeratin 18 (CK18) (Supplemental Figure 6, C–E), and CK18 knockdown diminished osteoclast activation (Supplemental Figure 6, F–H). These findings establish that LOs deliver CK18-bound circCCDC50 to promote osteoclastogenesis and drive bone metastasis in denosumab-resistant ER+ BC.

### CRKL promotes circCCDC50 biogenesis through the JNK/ATF-2/EIF4A3 axis.

**Figure 7:**
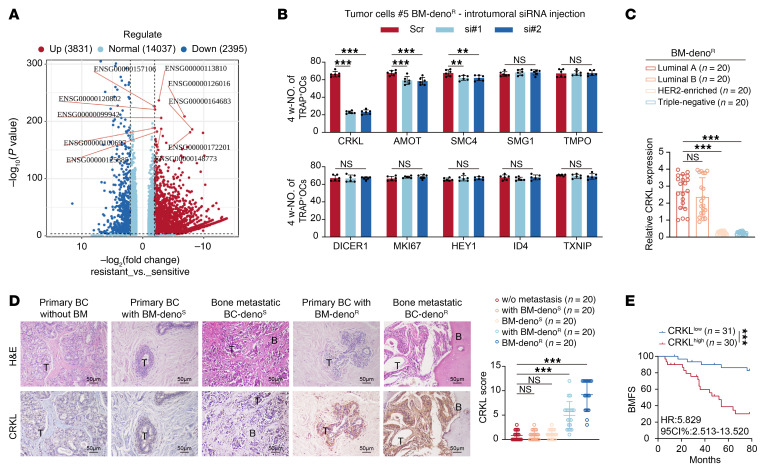
CRKL is upregulated in denosumab-resistant ER+ breast cancer cells and promotes bone metastasis. (**A**) Volcano plot of differentially expressed genes between denosumab-resistant and denosumab-sensitive ER+ BC cells identified by transcriptome sequencing. (**B**) TRAP+ multinucleated osteoclast counts in bone tissues of #5 BC models treated with dysregulated mRNA-targeting siRNA or nontargeting siRNA control (*n* = 6 mice/group). (**C**) The relative mRNA expression level of CRKL in BC tissues of different molecular subtypes was determined using qRT-PCR. (**D**) Left: H&E staining and representative immunohistochemical (IHC) staining of CRKL in breast tumor tissues and paired bone metastatic lesions from patients. Scale bars: 50 μm. Right: Quantitative IHC scoring revealed significantly elevated CRKL expression in bone metastases compared with primary tumors (*n* = 20 patients/group). (**E**) Kaplan–Meier analysis of bone metastasis-free survival curves in BC with high versus low expression of CRKL (*n* = 61). Each error bar represents the mean ± SD of 6 independent experiments. Significant differences were determined by 1-way ANOVA with Tukey’s multiple comparison test. *P < 0.05, **P < 0.01, ***P < 0.001.

**Figure 8:**
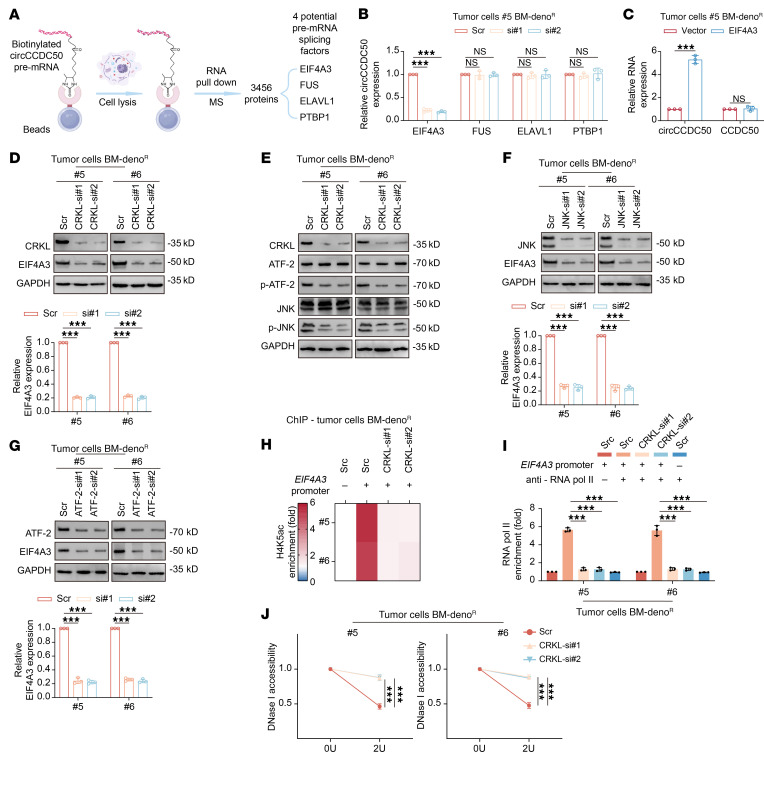
CRKL promotes circCCDC50 biogenesis through the JNK/ATF-2/EIF4A3 axis. (**A**) Schematic workflow of RNA pull-down assay combined with mass spectrometry to identify circCCDC50-binding cyclization factors following circRNA enrichment. (**B**) qRT-PCR analysis of circCCDC50 expression in indicated #5 primary tumor cells. GAPDH served as the loading control. (**C**) qRT-PCR quantification of circCCDC50 and linear CCDC50 mRNA levels in #5 primary tumor cells overexpressing EIF4A3 versus controls. (**D**–**G**) CRKL-mediated promotion of circCCDC50 biogenesis via the JNK/ATF-2/EIF4A3 signaling axis was validated by qRT-PCR and Western blot analyses. (**H**) H4K5ac enrichment at the EIF4A3 promoter region was elevated in resistant cells by ChIP-qPCR. EIF4A3 promoter (+) indicates the H4K5ac enrichment at the EIF4A3 promoter region; EIF4A3 promoter (–) indicates the enrichment at a negative control region. (**I**) RNA pol II occupancy at the EIF4A3 promoter was elevated in resistant cells by ChIP-qPCR. EIF4A3 promoter (+) indicates the RNA pol II enrichment at the EIF4A3 promoter region; EIF4A3 promoter (–) indicates the RNA pol II enrichment at a negative control region. anti-RNA pol II (+) indicates ChIP with an anti-RNA polymerase II antibody; anti-RNA pol II (–) indicates ChIP with a control IgG antibody. (**J**) DNase I hypersensitivity assay revealed reduced chromatin openness at the EIF4A3 promoter in CRKL-knockdown cells (#5, #6) compared with nonknockdown controls. Each error bar represents the mean ± SD of 3 independent experiments. Significant differences were determined by 1-way ANOVA with Tukey’s multiple comparison test. *P < 0.05, **P < 0.01, ***P < 0.001.

To investigate the mechanism underlying circCCDC50 enrichment in LOs from denosumab-resistant ER+ BC bone metastasis models, we performed transcriptome sequencing of BC specimens and identified 3,831 genes that were significantly upregulated in denosumab-resistant ER+ BC cells (Figure 7A). We further validated the expression of the top 10 most significantly upregulated genes in clinical BC specimens (Supplemental Figure 7A). In vivo functional screening demonstrated that *CRKL* knockdown most potently suppressed premetastatic osteoclastogenesis (Figure 7B and Supplemental Figure 7B). Clinically, *CRKL* was overexpressed in ER+ BC tumors (Figure 7C) and predicted shorter bone metastasis–free survival (Figure 7, D and E). Ectopic *CRKL* expression in sensitive models conferred resistance to denosumab-mediated osteoclast suppression (Supplemental Figure 7, C–E). Conversely, *CRKL* knockdown in resistant models restored sensitivity to denosumab treatment (Supplemental Figure 7, F and H). Analysis of data from the Kaplan–Meier plotter revealed a significant inverse correlation between *CRKL* expression and distant metastasis free survival (DMFS) of patients with ER+ BC patients who received systemic treatment (*n* = 1,141, *P* = 0.0093) (Supplemental Figure 8A). Overexpression of *CRKL* in denosumab-resistant ER+ BC cells did not significantly alter their malignant phenotype. Specifically, it had no marked effect on proliferative capacity in colony formation assays, nor on migratory or invasive potential in transwell assays (Supplemental Figure 8, B–D).Furthermore, RNA IP-mass spectrometry identified EIF4A3 as a direct circCCDC50-binding protein (Figure 8, A–C, and Supplemental Figure 8, E and F), while CRKL activated the JNK/ATF-2 axis to upregulate EIF4A3 and enhance circCCDC50 biogenesis (Figure 8, D–G, and Supplemental Figure 8, G–J). *CRKL* depletion significantly reduced *EIF4A3* promoter H4K5ac, H4K8ac, and H4K16ac enrichment. Among these, H4K5ac levels in the *EIF4A3* promoter were most elevated (Figure 8H and Supplemental Figure 8, K and L). However, no notable changes were observed in H3K9ac or H3K27ac levels (Supplemental Figure 8, M and N). *CRKL* depletion markedly reduced chromatin accessibility and RNA Pol II recruitment of *EIF4A3* promoter (Figure 8, I and J). Overexpression of *EIF4A3* in denosumab-sensitive ER+ BC cells significantly increased the level of circCCDC50, both intracellularly and within the secreted LOs (Supplemental Figure 9A). In the presence of denosumab, CM from *EIF4A3*-overexpressing, denosumab-sensitive bone metastatic cells (#3, #4) potently enhanced osteoclastogenesis, as shown by increased TRAP+ multinucleated cell numbers and enzymatic activity (Supplemental Figure 9, B and C). To assess the functional impact in vivo, we established orthotopic mammary tumors using the same cell lines. BLI at 8 weeks after resection revealed diffuse metastatic signals in the limbs. Quantitative μCT analysis showed that mice bearing *EIF4A3*-overexpressing tumors developed significantly larger osteolytic lesions than controls, independent of denosumab treatment. Concomitantly, histomorphometry confirmed a higher density of TRAP+ osteoclasts within the bone microenvironment of the *EIF4A3*-overexpressing group (Supplemental Figure 9, D–J). These findings establish CRKL as a master regulator of the EIF4A3-circCCDC50 axis that drives denosumab resistance in bone metastatic ER+ BC.

### The CircCCDC50/CARM1 complex enhances NFATc1 transcription to sustain osteoclastogenesis.

**Figure 9:**
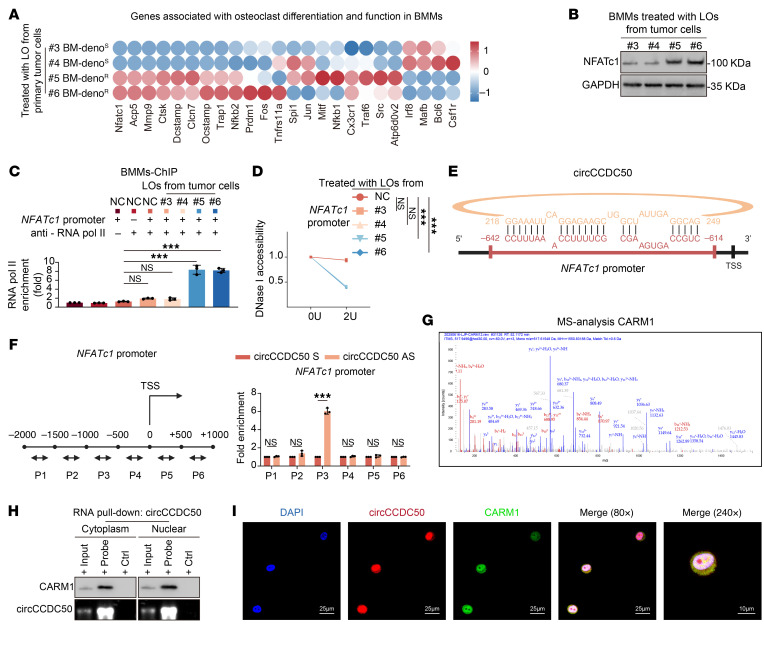
The circCCDC50/CARM1 complex enhances NFATc1 transcription to sustain osteoclastogenesis. (**A**) qRT-PCR analysis of transcriptional levels of osteoclast differentiation and activation-related genes in BMMs treated with conditioned media from distinct primary BC cells. (**B**) NFATc1 protein levels were evaluated in primary BC cells (denosumab-resistant lines #5, #6 and sensitive lines #3, #4). GAPDH served as a loading control. (**C**) RNA pol II occupancy at the NFATc1 promoter was elevated in BMMs treated with LOs derived from primary tumor cells by ChIP-qPCR. NFATc1 promoter (+) indicates the RNA pol II enrichment at the NFATc1 promoter region; NFATc1 promoter (–) indicates the RNA pol II enrichment at a negative control region; anti-RNA pol II (+) indicates ChIP with an anti-RNA polymerase II antibody; anti-RNA pol II (–) indicates ChIP with a control IgG antibody. (**D**) Resistant tumor-derived LOs significantly enhanced chromatin openness at the NFATc1 promoter. (**E**) Predicted circCCDC50-binding regions (–1,000 to –500 bp) on the NFATc1 promoter. (**F**) CHiRP-qPCR demonstrated significant enrichment of circCCDC50 at the NFATc1 promoter in resistant cells compared with scrambled probe controls. (**G**) Proteins coprecipitated with circCCDC50 were identified by ChIRP-MS. CARM1 (coactivator-associated arginine methyltransferase 1) exhibited high binding affinity. (**H**) RNA pull-down assay analysis of cytoplasmic and nuclear circCCDC50-interacting proteins (“probe” stands for circCCDC50 probe; “Ctrl” stands for control probe). (**I**) IF staining showed nuclear colocalization of circCCDC50 and CARM1 in BMMs treated with resistant tumor-derived LOs. Each error bar represents the mean ± SD of 3 independent experiments. Significant differences were determined by 1-way ANOVA with Tukey’s multiple comparison test. *P < 0.05, **P < 0.01, ***P < 0.001.

**Figure 10:**
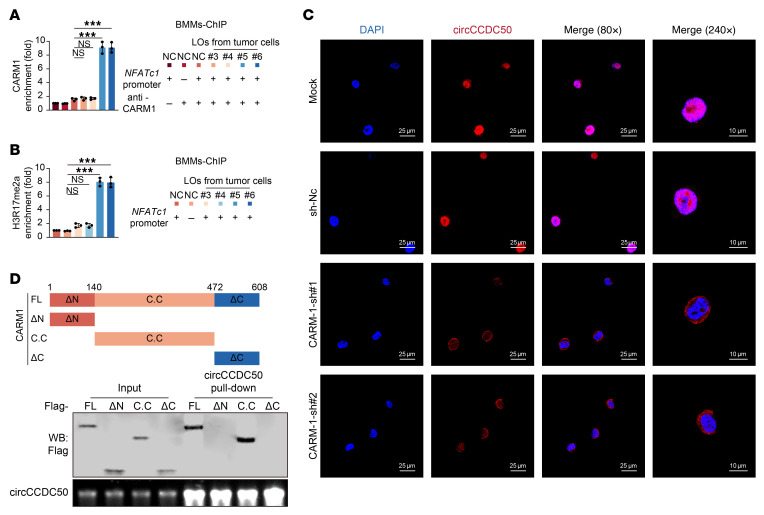
CARM1 is recruited by circCCDC50 to the NFATc1 promoter, where it mediates H3R17me2a enrichment. (**A**) CARM1 occupancy at the NFATc1 promoter was elevated in BMMs treated with LOs derived from primary tumor cells by ChIP-qPCR. NFATc1 promoter (+) indicates the CARM1 enrichment at the NFATc1 promoter region; NFATc1 promoter (–) indicates the CARM1 enrichment at a negative control region. anti-CARM1 (+) indicates ChIP with an anti-CARM1 antibody; anti-CARM1 (–) indicates ChIP with a control IgG antibody. (**B**) H3R17me2a enrichment at the NFATc1 promoter region was elevated in BMMs treated with LOs derived from primary tumor cells by ChIP-qPCR. NFATc1 promoter (+) indicates the H3R17me2a enrichment at the NFATc1 promoter region; NFATc1 promoter (–) indicates the enrichment at a negative control region. (**C**) Representative IF images of circCCDC50 (red) and CARM1 (green) in cells with or without CARM1 knockdown. (**D**) Upper: Schematic of CARM1 protein domains. Lower: Immunoblot (IB) validation of truncated CARM1 constructs confirming the intermediate domain (aa 200–400) as essential for circCCDC50 binding. Each error bar represents the mean ± SD of 3 independent experiments. Significant differences were determined by 1-way ANOVA with Tukey’s multiple comparison test. *P < 0.05, **P < 0.01, ***P < 0.001.

To elucidate the downstream mechanisms by which circCCDC50 sustains osteoclastogenesis despite denosumab treatment, we performed transcriptional analysis of osteoclast differentiation markers in bone marrow–derived monocytes (BMMs) cocultured with tumor conditioned medium. Osteoclast activation–related genes were upregulated in high-metastatic potential groups, regardless of denosumab sensitivity status. Specifically, *NFATc1* expression was markedly elevated in osteoclasts cocultured with denosumab-resistant ER+ BC (Figure 9, A and B). Furthermore, we demonstrated enhanced recruitment of RNA polymerase II to the *NFATc1* promoter region following exposure to denosumab-resistant ER+ BC cells (Figure 9C). This genomic locus concurrently exhibited increased chromatin accessibility, as evidenced by heightened DNase I hypersensitivity (Figure 9D). Ectopic overexpression of circCCDC50 in BMMs significantly elevated *NFATc1* expression levels (Supplemental Figure 10A). In denosumab-resistant ER+ BC cells, knockout of circCCDC50 significantly attenuated the ability of their derived LOs to induce the upregulation of *NFATc1* at both the mRNA and protein levels. Conversely, introduction of circCCDC50 into mouse BMMs significantly elevated the mRNA and protein levels of *NFATc1* (Supplemental Figure 10, B and C). ChIP-qPCR demonstrated that circCCDC50 directly bound the *NFATc1* promoter region (–1,000 to –500 bp) (Figure 9, E and F). Strikingly, CARM1 can also directly occupy the *NFATc1* promoter region (–1,000 to –500 bp) (Supplemental Figure 10D). Through comprehensive CHiRP-MS profiling, we identified the RNA-binding protein CARM1 as a direct interactor of circCCDC50 (Figure 9G). RNA pulldown combined with Western blot, as well as IF coupled with fluorescence in situ hybridization, demonstrated that circCCDC50 binds to CARM1 in both the cytoplasm and the nucleus (Figure 9, H and I). CircCCDC50 recruited CARM1 to mediate asymmetric dimethylation of histone H3R17, thereby enhancing *NFATc1* transcription (Figure 10, A and B). Knockdown of CARM1 significantly reduced asymmetric dimethylation of histone H3 arginine 17 (H3R17me2a) at the *NFATc1* promoter, concomitant with diminished RNA polymerase II occupancy at this locus (Figure 10C and Supplemental Figure 10, E and F). Furthermore, CARM1 depletion markedly decreased both mRNA and protein expression levels of *NFATc1* (Supplemental Figure 10, G and H). Importantly, CARM1 knockdown substantially impaired nuclear retention of circCCDC50 (Supplemental Figure 10I). Protein truncation assays reveal that circCCDC50 binds to the catalytic core domain of CARM1 (Figure 10D). Notably, osteoclast-derived factors were found to activate Smad4/PlexinB1 signaling in tumor cells, leading to CRKL phosphorylation and establishing a self-reinforcing CRKL-circCCDC50-NFATc1 signaling loop (Supplemental Figure 10, J–O).

### Pharmacological inhibition of CARM1 reduces persistent osteoclastogenesis and overcomes denosumab resistance.

**Figure 11:**
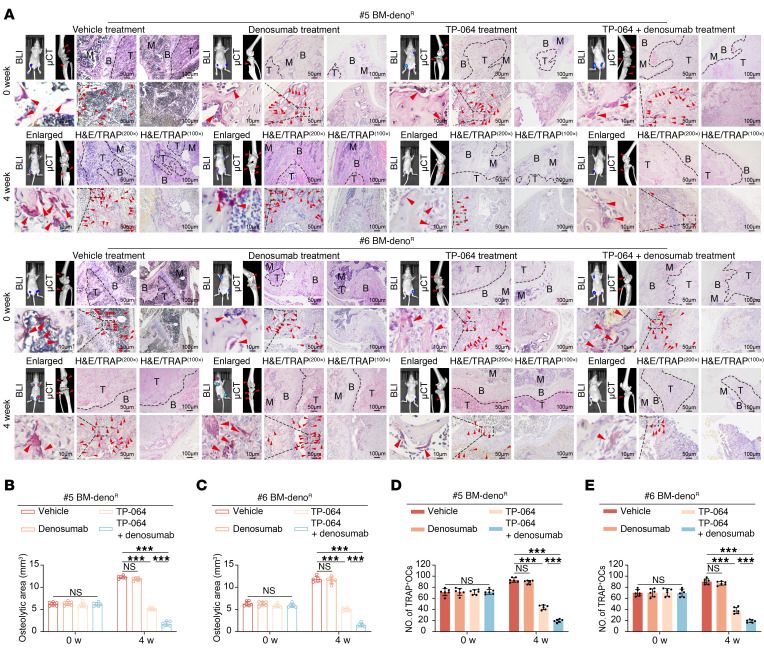
Therapeutic targeting of CARM1 inhibits denosumab-resistant bone metastasis in vivo. (**A**) BLI (left) and μCT (right) images and histological (H&E and TRAP) images (middle) of bone tissues from BC bone metastasis mouse models treated with indicated therapies. Scale bar: 100 μm (100×), 50 μm (200×), and 10 μm (Enlarged). (**B** and **C**) μCT-based analysis of osteolytic lesion area in bone tissues of BC bone metastasis mouse models treated with indicated therapies. (**D** and **E**) Quantification of TRAP+ multinucleated osteoclasts per field in bone tissues of BC bone metastasis mouse models treated with indicated therapies. Each error bar represents the mean ± SD of 6 independent experiments. Significant differences were determined by 1-way ANOVA with Tukey’s multiple comparison test. *P < 0.05, **P < 0.01, ***P < 0.001.

CARM1 inhibitor TP-064 treatment significantly suppressed osteoclast differentiation induced by LOs derived from denosumab-resistant ER+ BC cells (Supplemental Figure 11, A and B). Additionally, *NFATc1* upregulation in BMMs, mediated by denosumab-resistant ER+ BC cell-derived LOs, was significantly suppressed by TP-064 treatment (Supplemental Figure 11, C–F). TP-064 treatment ameliorated the trabecular bone structure damage in mice caused by LOs derived from denosumab-resistant ER+ BC cells and reduced the colonization of nonmetastatic ER+ BC cells in the bone microenvironment (Supplemental Figure 11, G-K). Treatment with TP-064 was initiated in mice once the bioluminescence signal of bone-metastatic tumors reached 2 × 10^6^ p/sec/cm²/sr. After 4 weeks of treatment, vehicle-treated mice exhibited a higher incidence of bone metastases, a greater bone-metastatic tumor burden, severe osteolytic bone lesions, and increased numbers of TRAP-positive osteoclasts along the bone-tumor interface (Figure 11, A–E).

**Figure 12:**
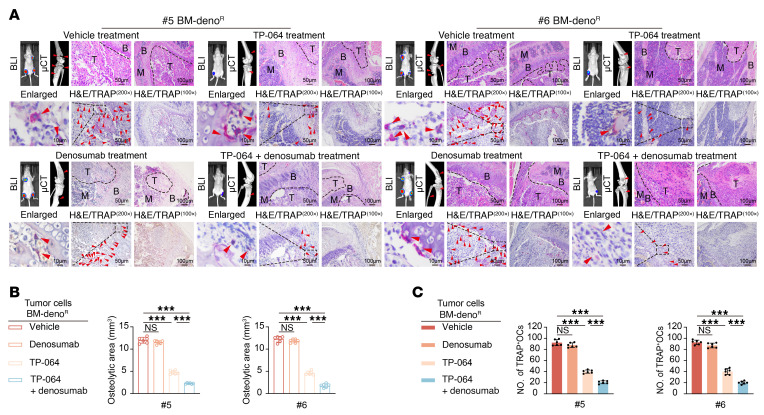
Early pharmacological inhibition of CARM1 by TP-064 attenuates the formation of denosumab-resistant bone metastases. (**A**) BLI (left) and μCT (right) images and histological (H&E and TRAP) images (middle) of bone lesions from mice, which received vehicle or TP-064 treatment starting at 2 days after intracardial injection of #5 or #6 cells. (**B**) Quantification of osteolytic sites along the bone-tumor interface of metastases from experiments in left and middle panels. Scale bar: 100 μm (100×), 50 μm (200×), and 10 μm (Enlarged). (**C**) Quantification of TRAP+ multinucleated osteoclasts per field in bone tissues. Scale bar: 100 μm (100×), 50 μm (200×), and 10 μm (Enlarged). Each error bar represents the mean ± SD of 6 independent experiments. Significant differences were determined by 1-way ANOVA with Tukey’s multiple comparison test. *P < 0.05, **P < 0.01, ***P < 0.001.

Furthermore, in a separate experiment, TP-064 treatment was initiated 2 days following the intracardiac injection of BM-Deno^R^ BC cells. Compared with vehicle treatment, TP-064 significantly delayed the development of bone metastasis and reduced both the incidence of bone metastases and the bone-metastasis burden (Supplemental Figure 11, L and M). Mice treated with TP-064 also exhibited smaller osteolytic areas and fewer TRAP-positive osteoclasts per bone surface area (Figure 12, A–C). Histopathological examination revealed that TP-064 administration caused no significant adverse effects on major organs (including the heart, liver, lung, kidney, and spleen) in tumor-free mice; moreover, it did not alter the trabecular bone structure (Supplemental Figure 11, N and O). The colony formation assay showed that TP-064 at a concentration of 0.1 μM did not significantly inhibit the growth of BC cells (Supplemental Figure 11P). ChIP assay data show that CARM1 inhibition by TP-064 significantly reduced the enrichment of H3R17me2 at the *NFATc1* promoter of BMMs in vivo (Supplemental Figure 11Q).

Our study reveals a mechanism of denosumab resistance in ER+ BC bone metastasis mediated by tumor-derived LOs. These LOs deliver CRKL-induced circCCDC50 to BMMs, where it recruits CARM1 to activate *NFATc1*, thereby driving RANKL-independent osteoclastogenesis. Importantly, CARM1 inhibition represents a promising therapeutic strategy for treating denosumab-resistant bone metastases.

## Discussion

Our study elucidates a previously unrecognized RANKL-independent molecular axis that drives denosumab resistance in ER+ BC bone metastasis. Mechanistically, tumor cells resistant to denosumab exhibit sustained osteoclast activation through a self-amplifying CRKL/circCCDC50/NFATc1 signaling cascade. A key clinical observation is the significant upregulation of CRKL transcription in ER+ BC cells from denosumab-resistant patients compared with responsive ones. This elevated *CRKL* expression acts as a persistent driver, leading to sustained activation of the JNK pathway, as evidenced by increased levels of phosphorylated JNK. Furthermore, we demonstrate that activated JNK directly phosphorylates and enhances the activity of the transcription factor ATF-2. This posttranslational modification enables ATF-2 to bind to the promoter of the *EIF4A3* gene, thereby promoting its transcription. The functional importance of CRKL at the apex of this cascade is confirmed by our loss-of-function experiments: CRKL knockdown significantly reduces the phosphorylation levels of both JNK and ATF-2, inhibits their sustained activation, and consequently diminishes *EIF4A3* transcription. EIF4A3, an essential RNA helicase and core component of the exon junction complex, is also a well-established facilitator of back splicing, the fundamental step in circRNA biogenesis. Thus, through this axis, CRKL ultimately licenses the increased biogenesis of circCCDC50, which is highly expressed in ER+ BC cells from patients who are denosumab resistant. This circular RNA is selectively encapsulated into LOs and delivered to bone marrow–derived macrophages. Within these recipient cells, circCCDC50 functions as a molecular scaffold, recruiting CARM1 to the *NFATc1* promoter. CARM1 catalyzes histone H3 arginine 17 (H3R17) dimethylation, creating an open chromatin configuration that facilitates *NFATc1* transcription. Elevated NFATc1 not only drives osteoclast differentiation and bone resorption but also feeds back to tumor cells, further amplifying CRKL expression. This vicious cycle sustains osteolytic progression even under RANKL blockade, explaining the clinical inefficacy of denosumab in this patient subset (10–13). Importantly, pharmacological inhibition of CARM1 using TP-064 disrupts this loop by suppressing asymmetric dimethylation of *NFATc1* promoter, as demonstrated in denosumab-resistant CDX models where TP-064 significantly inhibited bone metastasis progression. These findings position CARM1 as a potential therapeutic target for overcoming RANKL-independent resistance.

While the majority of reported circRNAs exert their functions in the cytoplasm, where they modulate mRNA stability, sponge miRNAs, regulate protein activity (25, 26), our discovery that circCCDC50 enters the nucleus to directly regulate *NFATc1* transcription represents an important expansion of circRNA biology. Previous studies have established that most circRNAs are retained in the cytoplasm due to their splicing-derived structure and lack of nuclear localization signals (NLS) (27). However, emerging evidence suggests that certain circRNAs can localize to the nucleus and participate in transcriptional regulation. For instance, circANRIL was shown to bind the ribosomal DNA (rDNA) locus and recruit the Polycomb repressive complex 2 (PRC2) to modulate rRNA maturation (28). Similarly, circEIF3J and circPAIP2 were found to interact with U1 small nuclear ribonucleoprotein (snRNP) and RNA polymerase II to enhance transcription of their parental genes (29). Our findings add to this growing body of evidence by demonstrating that circCCDC50 not only translocates to the nucleus but also recruits the epigenetic modifier CARM1 to the *NFATc1* promoter, thereby directly enhancing transcription. This mechanism is distinct from classical cytoplasmic circRNA functions and suggests that nuclear-localized circRNAs may play underappreciated roles in gene regulatory networks, particularly in pathological contexts such as cancer metastasis (30). The precise mechanism by which circCCDC50 enters the nucleus remains to be fully elucidated, but its association with CK18 in LOs may facilitate nuclear translocation, as CK18 has been implicated in nucleocytoplasmic shuttling (31). Future studies should explore whether other nuclear-localized circRNAs similarly regulate osteoclastogenesis or other bone remodeling processes.

Prior research on ER+ BC bone metastasis has predominantly focused on RANKL-dependent mechanisms. For instance, estrogen receptor signaling directly upregulates RANKL expression in tumor cells (32), while parathyroid hormone-related protein (PTHrP) and IL-11 secreted by tumor cells stimulate stromal cells (e.g., osteoblasts) to produce RANKL, indirectly promoting osteoclastogenesis (33, 34). While denosumab demonstrates efficacy in reducing SRE risk by 18% compared with bisphosphonates, nearly 40% of patients develop resistance to denosumab within 12 months, exhibiting aggressive bone metastasis progression (10, 12, 35). Recent studies implicate additional RANKL-enhancing pathways, such as C-X-C motif chemokine ligand 12 (CXCL12)/C-X-C chemokine receptor type 4 (CXCR4) axis-mediated osteoblast suppression (36) and Notch-induced Jagged1-RANKL crosstalk (37). However, therapeutic targeting of these pathways (e.g., with CXCR4 antagonists) has shown limited clinical benefit due to compensatory RANKL-independent escape mechanisms (38).

Emerging evidence suggests that RANKL-independent osteoclast activation plays a pivotal role in therapeutic resistance. For example, Wnt family member 5A (WNT5A) secreted by tumor cells activates β-catenin–independent signaling in osteoclast precursors via receptor tyrosine kinase-like orphan receptor 1 (ROR1), promoting differentiation even in RANKL-neutralized microenvironments (39). Similarly, transforming growth factor-β (TGF-β) released from bone matrix during early osteolysis activates SMAD family member 3 (SMAD3) in osteoclasts, inducing cathepsin K (CTSK) expression and resorption activity independently of RANKL (40). However, no clinically approved therapies directly target these pathways. Preclinical attempts to target WNT5A-ROR1 with monoclonal antibodies (e.g., vantictumab) or TGF-β inhibitors (e.g., galunisertib) have shown modest antiresorptive effects but failed to inhibit tumor progression (41, 42), likely due to tumor-intrinsic resistance mechanisms. This underscores the unmet need for strategies that concurrently disrupt tumor-bone crosstalk and tumor survival pathways (22). Our study identifies a RANKL-independent pathway, the CRKL/circCCDC50/NFATc1 signaling cascade, that promotes persistent progression of ER+ BC bone metastases and mediates denosumab resistance, providing mechanistic insights.

Clinically, the inability to predict denosumab resistance early remains a major challenge. The delayed identification of denosumab nonresponders leads to high SRE incidence, poor prognosis, and significantly diminished quality of life (43, 44). Current approaches rely on late radiographic indicators (e.g., CT-based lytic lesion progression) or biochemical markers (e.g., uNTX, tartrate-resistant acid phosphatase 5b; TRACP5b), which lack sensitivity (45). For instance, serum RANKL/osteoprotegerin (OPG) ratios exhibit instability and poor correlation with bone turnover in denosumab-treated patients (46). Furthermore, a critical challenge persists in clinical practice: a subset of patients with BC with bone metastases who are initially denosumab sensitive exhibit such aggressive disease progression that denosumab fails to protect against progressive bone destruction. These patients present with clinical symptoms, radiographic findings, and changes in conventional biomarkers that are indistinguishable from those with primary or acquired denosumab resistance, making it clinically difficult to differentiate between these 2 groups and implement precise therapeutic strategies. Our study provides a transformative insight into this dilemma. We discovered that circCCDC50 is specifically enriched in LOs derived from denosumab-resistant tumors. Correspondingly, plasma levels of circCCDC50 were significantly higher in resistant patients compared with sensitive ones, and its elevated expression correlated strongly with shorter bone metastasis–free survival. Crucially, as a circular RNA, circCCDC50 demonstrates remarkable resistance to ribonuclease degradation and exhibits exceptional stability in the bloodstream, a distinct advantage over conventional biomarkers like microRNAs (miRNAs) or proteins (47, 48). This intrinsic stability establishes its strong potential as an ideal liquid biopsy target. Consequently, plasma circCCDC50 levels hold promise as a stable and reliable biomarker for the early and accurate discrimination of true therapeutic resistance from cases of ultra-aggressive disease. Integrating circCCDC50 quantification into existing clinical assessment frameworks could refine patient risk stratification and ultimately guide personalized precision therapy (49). Therapeutic paradigms for denosumab-resistant cases have historically prioritized tumor-directed therapies while neglecting microenvironmental drivers of SREs. This oversight is critical, as up to 60% of mortality in advanced bone metastasis results from SRE-related complications (e.g., hypercalcemia, spinal cord compression) (5). Our study highlights the critical need to target both tumor cells and the bone microenvironment in denosumab-resistant ER+ BC. The CARM1 inhibitor TP-064 emerges as a promising therapeutic candidate by simultaneously disrupting 2 key pathological processes: (a) it abolishes NFATc1-driven osteoclastogenesis through epigenetic silencing of the NFATc1 promoter (reducing osteolytic lesions by 78% in preclinical models, *P* < 0.001), and (b) it interrupts the tumor-microenvironment feedback loop by blocking osteoclast-derived SEMA4D-induced CRKL activation in cancer cells (50). Notably, TP-064 maintains a favorable safety profile due to its high selectivity for CARM1 (51), which preserves normal bone remodeling while avoiding the off-target effects associated with broader epigenetic modifiers. TP-064 represents a clinically translatable strategy for patients progressing on RANKL inhibitors (52), particularly those with high circCCDC50 levels who may derive greatest benefit.

In conclusion, our work found a feedforward CRKL/circCCDC50/NFATc1 axis that induced RANKL-independent osteolysis in ER+ BC, leading to denosumab resistance. Future studies should validate circCCDC50-guided therapeutic algorithms and the potential application of CARM1 inhibitor TP-064 in patients with ER+ BC in prospective clinical trials. These advances may transform the management of patients with denosumab-resistant BC bone metastasis, ultimately improving survival and quality of life for this vulnerable population (53, 54).

## Methods

### Sex as a biological variable.

All participants were female, reflecting the sex-specific demographics of BC. Accordingly, only female mice were used for orthotopic and metastatic tumor models.

### Patient samples in experiments.

This study complied with all relevant ethical regulations and included 247 breast tumor samples and 224 peripheral blood samples. The tumor cohort comprised 207 primary BC tissues (56 nonmetastatic BC, 48 denosumab-sensitive bone metastatic BC, and 103 denosumab-resistant bone metastatic BC) and 40 bone metastatic BC tissues (at bone), all histopathologically and clinically diagnosed at Sun Yat-sen Memorial Hospital, Sun Yat-sen University between 2020 and 2025. Peripheral blood samples included 145 from the above patients and 79 from additional patients with primary BC. Clinical characteristics of patients whose BC cells were used to establish CDX models are detailed in Supplemental Table 1. The study protocol was approved by the Institutional Research Ethics Committee of Sun Yat-sen University, permitting use of these clinical materials for research purposes. All samples were collected in accordance with the Declaration of Helsinki, and written informed consent was obtained from each patient.

### Collection of breast in situ tumor tissue specimens for high-throughput transcriptome sequencing.

After tissue collection, samples were sent to the Sangon Biotech (Shanghai, China) Co., Ltd. for high-throughput sequencing. Key procedures included: RNA Extraction, Library Construction and Sequencing, Filtering of Clean Reads, Alignment with Ribosome RNA (rRNA), Alignment with Reference Genome, Quantification of Gene Abundance, Correlation Analysis of Replicas, and Principal Component Analysis.

### Cell lines.

Primary cell lines #1–#6 were established in our lab. HEK293T cells were sourced from the American Type Culture Collection (ATCC). All cell lines were cultured following standard protocols.

### Tumor tissue processing.

Fresh patient-derived tissue samples were briefly stored in MACS Tissue Storage Solution (Miltenyi Biotec, 130-100-008) to maintain viability prior to processing. In a sterile biosafety cabinet, samples were dissected using sterile scissors and scalpels to remove adipose and necrotic tissues. Portions of the tissue were cryopreserved at –80°C for subsequent RNA extraction and total protein isolation to assess gene expression. Another portion was fixed in 4% paraformaldehyde for 24–48 hours, followed by paraffin embedding and sectioning for H&E staining and IHC analysis.

### Humanization of mouse model.

Female NCG-hairless mice (GemPharmatech, T003257), purchased from the GemPharmatech Co., Ltd, were raised in the SPF animal facility in the Laboratory Animal Resource Center at Sun Yat-Sen University. Specifically, mice were irradiated with a sublethal dose (1.0 Gy) and then injected intravenously with 2 × 10^4^ human umbilical cord blood-derived CD34+ cells. Subsequently, the NCG-hairless mice would develop a human immune system, including macrophages.

### Intracardiac injection.

NCG hairless mice (T003257 GemPharmatech Co., Ltd) were raised in the SPF animal facility in the Laboratory Animal Resource Center at Sun Yat-Sen University. After mouse humanization, primary tumor cell #1/#2 w/o metastasis BC cells (5 × 10^5^ cells in 100 μL PBS) were injected into the left cardiac ventricle.

### BLI.

Bone metastases were detected and quantified weekly after injection by BLI using IVIS 200 Spectrum (Revvity, USA). Mice were i.p.-injected with 150 mg/kg d-luciferin (MCE, HY-12591A) and anesthetized with 3% isoflurane 10 minutes before imaging.

### Drugs.

Mice received denosumab (MCE, HY-P9958) or its isotype control via subcutaneous injection in 100 μL PBS, twice weekly beginning at day 14 post-inoculation. Injections were performed using 29G insulin syringes (BD, 324826), with injection sites alternated between the left and right flank regions. TP-064 (MCE, HY-114965) was dissolved in DMSO and subsequently diluted in PBS to a final concentration of 2% DMSO. It was administered via intraperitoneal injection at a dose of 2.5 mg/kg, 9 times for a duration of 4 weeks. 29G insulin syringes were used, and injection sites were alternated between the left and right lower abdominal quadrants. EZM 2302 (MCE, HY-111109) was administered via oral gavage at a dose of 150 mg/kg, twice daily for 4 weeks. The compound was formulated in a vehicle of PBS containing a final concentration of 2% DMSO, with a typical administration volume of 100–200 μL per dose. Recombinant OPG-Fc (MCE, HY-P71017) was reconstituted in sterile PBS. It was administered via subcutaneous injection at a dose of 4 mg/kg, once weekly. The injection volume was 100 μL, using 29G syringes with alternating flank sites.

### H&E staining.

Sections were deparaffinized in xylene, rehydrated, and stained with Harris hematoxylin (5 min) and eosin (1 min). After dehydration and mounting, images were acquired using a Nikon ECLIPSE Ni-U.

### Osteoclastogenesis assay.

The mouse bone marrow cells (1 × 10^5^) were cultured on 24-well clusters containing glass coverslips (Thermo Fisher Scientific, USA) and grown in the conditioned media (CM). Media were changed every other day. Osteoclasts were counted on day 6. The osteoclasts cultured on plastic dishes were fixed with 4% paraformaldehyde/PBS (pH 7.4), and TRAP expression was examined by staining with a commercial kit (Sigma-Aldrich, 387A-1KT). Osteoclasts were defined as TRAP-positive multinucleated cells containing more than 3 nuclei.

### ALP staining.

TRAP/ALP Stain Kit (Takara, MK300) was used to perform ALP staining, according to the manufacturer’s instructions. The cells cultured in 24-well plates were rinsed 3 times by PBS (pH 7.4) and fixated for 30 min using prechilled fixative. Next, ALP substrate solution was added, and cells were cultivated (room temperature, 15–45 min) in a darkroom. After that, cells with ddH2O were rinsed and filmed under an optical microscope. The absorbance at 405 nm of each well was measured with a microplate reader according to the manufacturer’s instruction.

### IHC.

IHC was performed by using an SP Rabbit and Mouse HRP Kit (Cwbio, CW2069S), according to the manufacturer’s protocol. IHC was performed on paraffin sections of BC tissues or animal tumors using anti-CrkL antibody (Abcam, ab151791).

### IF staining.

Cells were rinsed briefly with PBS (pH 7.4) and fixed in 4% (w/v) paraformaldehyde in PBS (pH 7.4) for 20 min at 37°C. We aspirated fixation solution and washed cells 2–3 times in PBS (pH 7.4) followed by the anti-CARM1 antibody (Abcam, ab307091). The secondary antibody was goat anti-rabbit IgG (H + L) conjugated with Alexa Fluor 594 (Thermo Fisher Scientific, A-11012), or we added 1:1,000 dilution of Phalloidin-iFluor 488 (Abcam, ab176753) in 1% BSA (Thermo Fisher Scientific, #PI37537) at room temperature for 20–90 minutes and rinsed cells 2–3 times with PBS (pH 7.4) (5 min/wash). Then cells were mounted with Antifade Mountant with DAPI (Thermo Fisher Scientific, D1306). The images were captured using the Leica SP8 STED 3X.

### Measurement of human RANKL in mouse serum by ELISA.

Blood samples were collected from the retro-orbital plexus of anesthetized mice at indicated time points. Following coagulation for 30 minutes at room temperature, samples were centrifuged at 2,000*g* for 15 minutes at 4°C to isolate serum, which was then aliquoted and stored at –80°C until analysis. The concentration of human RANKL (huRANKL) in the serum was quantified using a commercially available human-specific RANKL ELISA kit (Abcam, ab213841) according to the manufacturer’s instructions. All samples and provided standards were assayed in duplicate. Briefly, serum samples were diluted appropriately with the provided assay diluent, added to the antibody-coated wells, and incubated. After washing, a biotinylated detection antibody was added, followed by streptavidin-HRP and a TMB substrate solution. The reaction was stopped with sulfuric acid, and the absorbance was measured at 450 nm (with a reference wavelength of 570 nm or 630 nm) using a microplate reader. The concentration of huRANKL in each sample was determined by interpolating the mean absorbance value against the standard curve generated from the known concentrations of the recombinant human RANKL standard.

### Establishment of bone organoid model.

The bone organoid model was established using a modification of the method as previously reported. In brief, 1.0 × 10^4^ MSCs were seeded on demineralized bone matrix in a 24-well plate and cultured with differentiation medium for 10 days. The ALP staining and alizarin red mineral staining were, respectively used to characterize differentiation and mineralization by MSCs derived osteoblasts in osteogenic differentiation medium on day 10. Next,1.0 × 10^5^ HSCs were added to each well and induced by 30 ng mL−1 MCSF (R&D Systems, 416-ML) for 14 days. In addition, TRAP staining and scanning electron microscopy were, respectively used to characterize differentiation and resorption pits were observed by HSCs-derived osteoclasts after coculture for day 14.

### RNA isolation and qRT-PCR.

The total RNA of BC cells was extracted using Trizol reagent (Invitrogen, USA) and the total RNA of BC tissues was extracted using Tissue RNA Purification Kit Plus (ES Science, China) according to the manufacturer’s instruction. Quantitative Real-time reverse transcription-polymerase chain reaction (PCR) primers and probes were designed with the assistance of the Primer Express v 2.0 software (Applied BioSystems, USA). The real-time quantitative polymerase chain reaction (qRT-PCR) was performed using SYBR qRT-PCR Master Mix (Vazyme Biotech, China) on Roche LightCycler 480 II system (Roche, Switzerland).

### Western blotting analysis.

Tumor cells were immersed in RIPA buffer for lysis. Lysates were then subjected to centrifugation at 15,000 rpm for 45 min at 4°C to obtain the protein lysates in the supernatant. Cells were lysed in RIPA buffer (Thermo Fisher Scientific, 89900). The concentration of protein was detected by bicinchoninic acid (BCA) kit (Cwbio, CW0014S). Around 30 g protein was separated by 7.5%–12.5% SDS-PAGE and then transferred onto PVDF membrane (Millipore, Germany). The PVDF membranes were blocked with 5% bovine serum albumin (BSA) at RT for 1 hour and then incubated with anti-GAPDH (Cell Signaling Technology, 8884), anti-CrkL antibody (Abcam, ab151791), anti-CARM1 antibody (Abcam, ab307091), anti-JNK1 + JNK2 + JNK3 antibody (Abcam, ab179461), anti-JNK1 + JNK2 + JNK3 (phospho T183+T183+T221) (Abcam, ab124956), overnight at 4°C respectively. A secondary antibody (Cell Signaling Technology, Anti-Rabbit:7074S, Anti-Mouse:7076S) was incubated for 1 hour at RT. Finally, the blots were detected by chemiluminescence kit (Millipore,WBKLS0500) and analyzed by Image Lab.

### Extracellular vesicle isolation from cultured BC cells.

Cells-conditioned culture medium was collected from approximately 95% confluent BC cells grown for 72 hours in 40 × 100 mm cell culture dishes with DMEM containing FBS depleted of bovine serum extracellular vesicles (EVs) by 24h ultracentrifugation at 120,000*g*, 4°C. The collected media was subjected to a centrifugation step of 400*g* for 10 min at room temperature (RT) to pellet and remove cells. All following centrifugation steps were performed at 4°C. Next, the supernatant was spun at 2,000*g* for 20 min to remove debris and apoptotic bodies. To pellet and collect large oncosomes (LOs), the supernatant was transferred into a centrifuge tube and centrifuged at 8,000*g* for 30 min to pellet LOs, which was resuspended in a large volume of phosphate-buffered saline (PBS) following ultracentrifugation at 8,000*g* for 30 min to precipitate LOs for further qNano analysis using NP2000nm nanopore. To remove any LOs’ contamination, the remaining supernatant was centrifuged at 10,000*g* for 30 min and transferred into a centrifuge tube for further centrifuging 40 min at 15,000*g*, and then the pellet was resuspended in a large volume of phosphate-buffered saline (PBS) following ultracentrifugation at 15,000*g* for 40 min to precipitate microvesicles (MVs) for further qNano analysis using NP800nm nanopore. To remove any remaining MVs, the media supernatant from the 15,000*g* step was passed through a 0.22 mm pore polyethersulfone (PES) filter (Millipore, USA). This supernatant (pre-cleared medium) was next subjected to ultracentrifugation at 120,000*g* for 4 hours in a SW32 Ti Rotor Swinging Bucket rotor (Beckman Coulter, CA) to sediment small extracellular vesicles (sEVs) for further qNano analysis using NP100nm nanopore. The crude sEV pellet was resuspended in a large volume of PBS followed by ultracentrifugation at 120,000*g* for 4 hours to wash the sample. At no time during the process were samples subjected to temperatures below 4°C.

### Large oncosome uptake assay.

Mouse bone marrow mononuclear cells were stained with Wheat Germ Agglutinin (WGA) conjugated to Alexa Fluor 488 (Thermo Fisher Scientific, W11261) to label the cell membrane. Separately, large oncosomes were labeled with the lipophilic membrane dye DIL (Thermo Fisher Scientific, D3911). Labeled large oncosomes were then incubated with the WGA-stained cells. After incubation, cells were washed thoroughly with PBS to remove unbound vesicles. Cellular uptake was assessed by fluorescence microscopy or flow cytometry, where the internalization of DIL-red fluorescent oncosomes into the WGA-green stained cell population was analyzed.

### Cytokine antibody array.

By using human Cytokine Antibody Array (Raybio, AAH-CYT-G5) according to the manufacturer’s instructions, cytokines were detected in media of #3 BM-Deno^S^ and #5 BM-Deno^R^. Membranes were blocked with the blocking buffer for 45 min, then incubated with 1 mL of samples containing protease inhibitor cocktail overnight at 4°C. After biotin-conjugated antibody and HRP-streptavidin incubation, chemiluminescence detection was performed.

### ELISA detection.

Peripheral blood samples from mice were collected and processed to obtain plasma or serum. ELISA detection was performed according the protocol. ELISA plates were coated with capture antibody overnight at 4°C, then blocked with a blocking buffer. Samples and standards were added to the wells, followed by detection antibody incubation. After washing, substrate solution was added for color development, and the reaction was stopped. Absorbance was measured, and data were analyzed to determine the concentration of the analyte in the samples.

### In vitro RNA transcription, circularization, and purification.

Linear RNAs were synthesized by in vitro transcription (IVT) from a linearized plasmid DNA template with the T7 polymerase promoter using a RiboMax Express Large Scale RNA Production system (Promega, P1320). After PCR amplification, 1 μg T7-DNA fragments were incubated with 0.5 mM dNTPs and 2 μl T7 RNA polymerase enzyme. Next, 2 mM GMP was introduced into the reaction mixture to phosphorylate the transcript at its 5′ end. IVT was carried out for 2 h at 37°C. After IVT, reactions were treated with DNase I (NEB, M0303S) for 15 min. The transcribed RNAs were ethanol-precipitated, followed by washing with 75% ethanol and finally resuspended in RNase-free water.

For in vitro circularization, a 20-nucleotide guide DNA oligomer acting as a template to link both ends of the linear RNA was designed and prepared. Next, 50 μg linear RNAs was incubated overnight at 16°C with T4 RNA ligase 1 (NEB, M0204L) within a 500 μl reaction volume. After ethanol precipitation, the RNAs were separated on denaturing urea polyacrylamide gel, followed by ethidium bromide staining and visualization. Circular or linear RNA bands were excised for RNA purification and further validated by RNase R treatment.

### CRISPR-Cas9 knockout system.

circCCDC50 knockout in BC cells was established using the CRISPR/Cas9 system as previously described (55, 56). Briefly, we designed single guide RNAs (sgRNAs) using an online tool (http://crispr.mit.edu/) and cloned them into the lenti-Cas9-eGFP vector (Addgene, 63592). Next, HEK293T cells were transduced with the lenti-Cas9-eGFP-sgRNA as well as pVSVg (Addgene, 8454) and psPAX2 (Addgene, 12260) to produce lentivirus. The viruses were concentrated with PEG5000 (Sigma Aldrich, Germany) and were used to infect BC cells. For circCCDC50, 2 sgRNAs flanking the target sequence were used at the same time. Next, GFP^+^ cells were isolated by FACS, followed by monoclonalization. Their deletion was verified by DNA sequencing.

### RNA fluorescence in situ hybridization.

Cy3-labeled circCCDC50 probes were designed and synthesized by RiboBio. A fluorescence *in situ* hybridization (FISH) kit (RiboBio, China) was used to detect the probe signals in BC cells according to the manufacturer’s instructions. Nuclei were stained with DAPI. All images were captured using the AxioVision Rel.4.6 computerized image analysis system (Carl Zeiss, Germany).

### RNase R/Actinomycin D.

Total RNA (2 μg) was incubated with 3 U/μg RNase R (Epicentre Technologies, USA) for 15 min at 37°C. BC cells were transferred to 6-well plates and treated with 5 μg/ml actinomycin D when the number of cells reached 9 × 10^5^, with samples collected at the indicated time points. The expression of circCCDC50 and the linear counterpart mRNA CCDC50 was analyzed by qRT-PCR.

### Chromatin isolation by RNA purification.

CircCCDC50 antisense probe was designed at the back-spliced site. All probes were synthesized with BiotinTEG at the 3′ end. The sequences of the probes were 5′-GTAGCTCAAGATGAATATGC-3′ (antisense probe) and 5′-GCATATTCATCTTGAGCTAC-3′ (sense probe).

### RNA pulldown assay.

The biotin-labeled circCCDC50 or control probe was incubated with streptavidin magnetic beads at room temperature for 1 h. Then the BC cell lysates were incubated with probe-beads complex at 4°C overnight for the binding of RNA-associated proteins to RNA. Subsequently, the RNA-protein complexes were washed 3 times and eluted from beads. The eluted proteins were finally analyzed by mass spectrometry or Western blot.

### RNA immunoprecipitation.

A Magna RNA binding protein immunoprecipitation kit was purchased from Millipore, and the experiment was conducted according to the manufacturer’s instructions. In brief, a total of 5 × 10^7^ cells were harvested and lysed using RIP lysis buffer. RNA-binding protein was immunoprecipitated using an anti-EIF4A3 specific antibody (Abcam, ab180573), and the retrieved RNA was subjected to qRT-PCR analysis. An anti-IgG antibody was used as a negative control. For the qRT-PCR analysis, circCCDC50 pre-mRNA Intron 1 was used as a nonspecific control, and lncRNA H19 was used as a positive control.

### ChIP assay.

The entire procedure was performed with the ChIP-IT Express Enzymatic (Active Motif, #53009) according to the manufacturer’s instructions. Briefly, indicated cells were grown to 70%–80% confluence on 100-mm culture dish and were fixed with 1% formaldehyde to cross-link proteins to DNA. The cell lysates were sonicated to shear DNA into small uniform fragments. Equal aliquots of chromatin supernatants were then immunoprecipitated overnight at 4°C using anti-CARM1 (Immunoway, YT7982) and anti-RNA pol II (Proteintech, AB_2732926) with protein G magnetic beads. The cross-linked protein/DNA complexes were collected by magnetic pull down, then were eluted from beads by elution buffer. After reverse cross-link of protein/DNA complexes to free DNA, PCR was performed using specific primers. We used Human Negative Control Primer Set 1 (Active Motif, #71001) and Human Negative Control Primer Set 1 (Active Motif, #71011) as negative control primers.

### Mass spectrometry.

Proteins were separated by SDS-PAGE and silver stained. Mass spectrum analysis was then performed to find potential binding proteins. The gel bands were separated and digested by gel electrophoresis and enzymes. Sequentially, the peptides were extracted by acetonitrile, dissolved in 0.1% formic acid, and delivered onto a tandem self-packed C18 column. The liquid elution was prepared for mass spectrometry with an Orbitrap Fusion (Thermo, USA) at BGI Co. Ltd (Shenzhen, China). Mascot (v2.3) software and Proteome Discoverer 3.1 were used to analyze the raw file and search the UniprotKB database to identify the proteins.

### Lentiviral preparation and transduction.

For lentiviral preparation, HEK-293T (5 × 10^5^) were transfected with target plasmids, 10 µg psPAX2 and 5 µg pMD2.G for 24 hours. After 24 hours culture, cells were incubated with fresh culture medium for another 48 hours. The viral supernatant was collected and incubated with BC cells, which are then selected by puromycin. The transfection efficiency was further analyzed by qRT-PCR.

### Statistics.

All data were presented as the mean ± SD. *n* represents the number of independent experiments performed on different mice or different batches of cells or different clinical tissues. Statistical analysis was performed using the Student’s 2-tailed *t* test and 1-way ANOVA. Bivariate correlations between study variables were calculated by Spearman’s rank correlation coefficients. Survival curves were plotted by the Kaplan–Meier method and compared by the log-rank test. The significance of various variables for survival was analyzed by univariate and multivariate Cox regression analyses. *P* values of 0.05 or less were considered statistically significant. Statistical analysis was performed using the GraphPad Prism 7.

### Study approval.

The present study was conducted in accordance with the guidelines of the Declaration of Helsinki, Belmont Report, and US Common Rule. The study protocol was approved by the Institutional Research Ethics Committee of Sun Yat-sen University, permitting use of these clinical materials for research purposes. All samples were collected in accordance with the Declaration of Helsinki, and written informed consent was obtained from each patient.

### Data availability.

MS proteomics data from this study have been deposited in the iProX database (https://www.iprox.cn/page/SCV017.html?query=IPX0015408000) under accession number IPX0015408000. The transcriptome and circRNA sequencing data discussed in this publication have been deposited in the NCBI Gene Expression Omnibus (GEO) and are accessible via the following GEO Series accession links: GSE317129 (https://www.ncbi.nlm.nih.gov/geo/query/acc.cgi?acc=GSE317129) and GSE317130 (https://www.ncbi.nlm.nih.gov/geo/query/acc.cgi?acc=GSE317130). The values for all data points in the graphs are reported in the Supporting Data Values file. The supporting data for the findings of this study are available from the corresponding authors upon request.

## Author contributions

The order of the three co–first authors reflects the relative scope and weight of their contributions to this manuscript. Qun Lin was placed first because she was responsible for conducting the core experiments, performing the primary data analysis, and leading the writing of the manuscript. Jinpeng Luo was placed second as he made substantial contributions to the experimental design and statistical methodology, which provided the essential framework for the study, while participating in figure preparation and manuscript writing. ZD was placed third for providing critical in vivo animal model data that validated the clinical relevance of the findings. All three authors have reviewed and approved this order. Jieer Luo assisted in data collection, statistical analysis, and figure preparation. WZ and YX performed experiments and analyzed data. YZ contributed to specimen collection and experimental procedures. XF interpreted data and edited the manuscript. J Liang participated in data acquisition and preliminary analysis. JC, Qianchong Lin, YQ, and RH assisted in experiments and data collection. HL and Q Liu provided technical support and experimental design input. J Li and CG supervised the project, provided critical revisions, and secured funding.

## Conflict of interest

The authors have declared that no conflict of interest exists.

## Funding support

- The Noncommunicable Chronic Diseases-National Science and Technology Major Project (2023ZD0501100).
- Shenzhen Medical Research Fund (B2502006).
- Natural Science Foundation of China (82371739, 82330082, 82072907).
- Science and Technology Projects in Guangzhou (2025A04J7152).
- The Science and Technology Planning Project of Guangdong Province (2023B1212060013).
- The Fundamental Research Funds for the Central Universities, Sun Yat-sen University (2022005).
- High-tech, Major and Characteristic Technology Projects in Guangzhou Area (2023-2025) (2023P-ZD14).
- Guangdong Provincial Clinical Research Center for Breast Diseases (2023B110005).
- Breast Cancer Clinical Research and Treatment Optimization Public Welfare Project (KYJJ20240509).
- Shanghai Anti-cancer Foundation (EBC-L002).

## Supplementary Material

Supplemental data

Unedited blot and gel images

Supporting data values

## Version 1 05/15/2026

Electronic publication
