# Supplementary material for: Targeting RANKL-independent osteoclastogenesis overcomes denosumab resistance in models of ER+ breast cancer bone metastasis
Source: J Clin Invest. 2026 May 15;136(10):e199285. doi: 10.1172/JCI199285 (PMC13178652; doi:10.1172/JCI199285)
Supplement: Supplemental data [file jci-136-199285-s310.pdf]

Supplementary figure 1

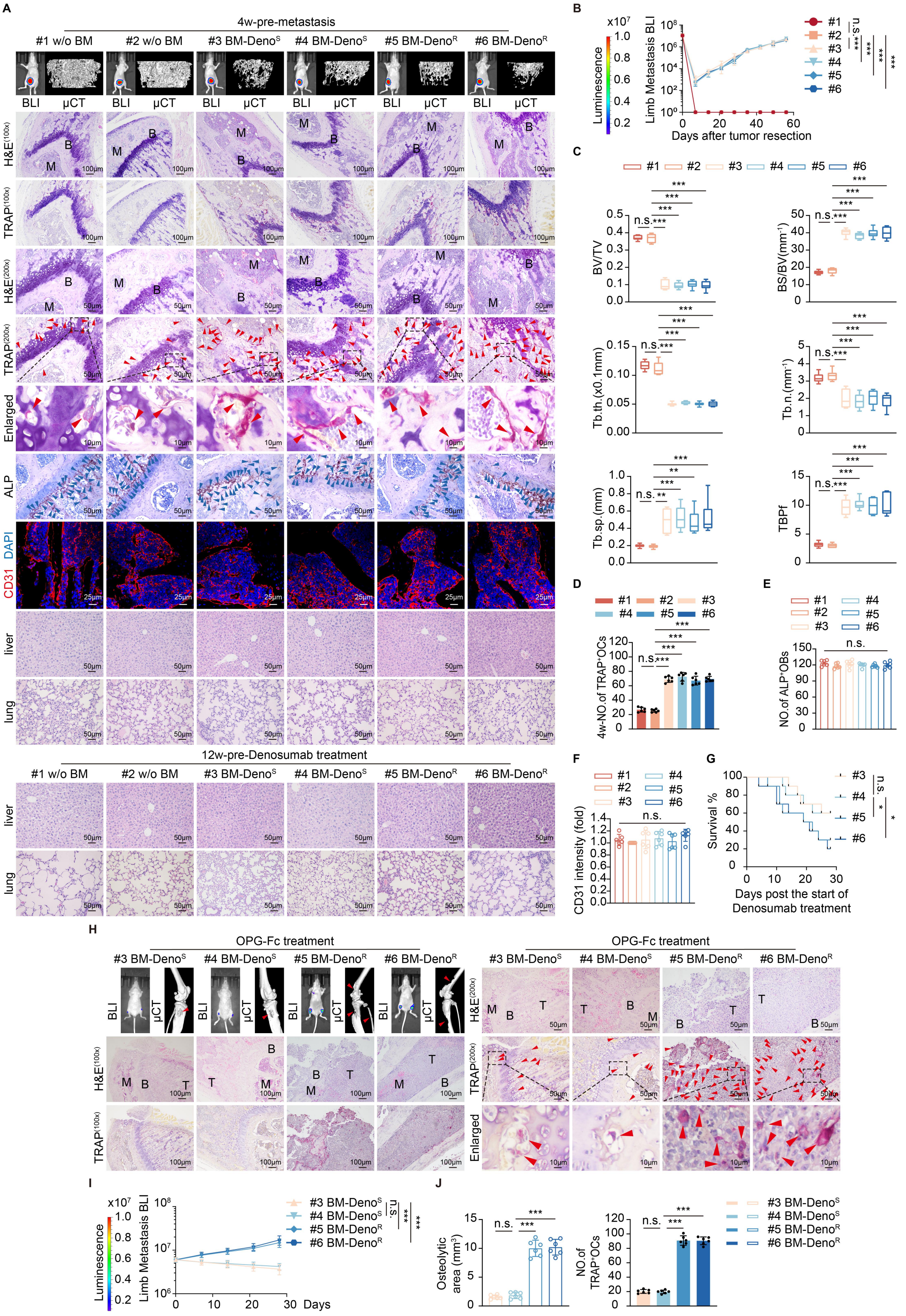

Supplementary figures and figure legends

Supplementary figure 1. Bone-metastatic breast cancer induces the continuous

activation of osteoclasts in the bone microenvironment before the occurrence of bone metastasis

A) Representative BLI images from breast cancer bone metastasis mouse models. representative H&E-stained sections of bone, liver and lung tissues from breast cancer bone metastasis mouse models. Representative μCT images from breast cancer bone metastasis mouse models. Representative TRAP-stained, ALP-stained, CD31 IF-stained sections of bone, tissues from breast cancer bone metastasis mouse models. Scale bar: 100 μm, 50 μm, 25μm and 10 μm.

B) Quantification of normalized BLI signals of bone metastases.

C) Quantification of the indicated bone parameters and from representative mice (n = 6 per group). BV/TV, bone/tissue volume ratio; BS/TV, bone surface/tissue volume ratio; Tb. n, trabecular number; Tb. sp., trabecular separation; Tb. th., trabecular thickness; TBPf, trabecular bone pattern factor.

D) Quantification of the indicated bone parameters and number of TRAP+ osteoclastss from representative mice

E) Quantification of number of ALP+ osteoblasts**.**

F) Quantification of CD31 fluorescence intensity (angiogenesis).

G) Kaplan-Meier curves showing prolonged survival in Denosumab-sensitive models (breast cancer bone metastasis mouse model#3-4) versus resistant models (breast cancer bone metastasis mouse model#5-6) after Denosumab therapy initiation.

H) BLI images of mice, μCT images of trabecular bone structure in the knee joint, Hematoxylin and eosin (H&E) staining of murine bone tissue, tartrate-resistant acid phosphatase (TRAP) staining. Scale bar: 100 μm, 50 μm and 10 μm.

I&J) Quantification of bone parameters, including normalized BLI signals of bone metastases (I), μCT-based osteolytic lesion area and $TRAP^{+}$ osteoclast numbers (J) in representative mice (n = 6 per group).

Each error bar represents the mean ± SD of 6 independent experiments. Significant differences were determined by one-way ANOVA with Tukey's multiple comparison test. * $p < 0.05$, ** $p < 0.01$, *** $p < 0.001$.

Supplementary figure 2

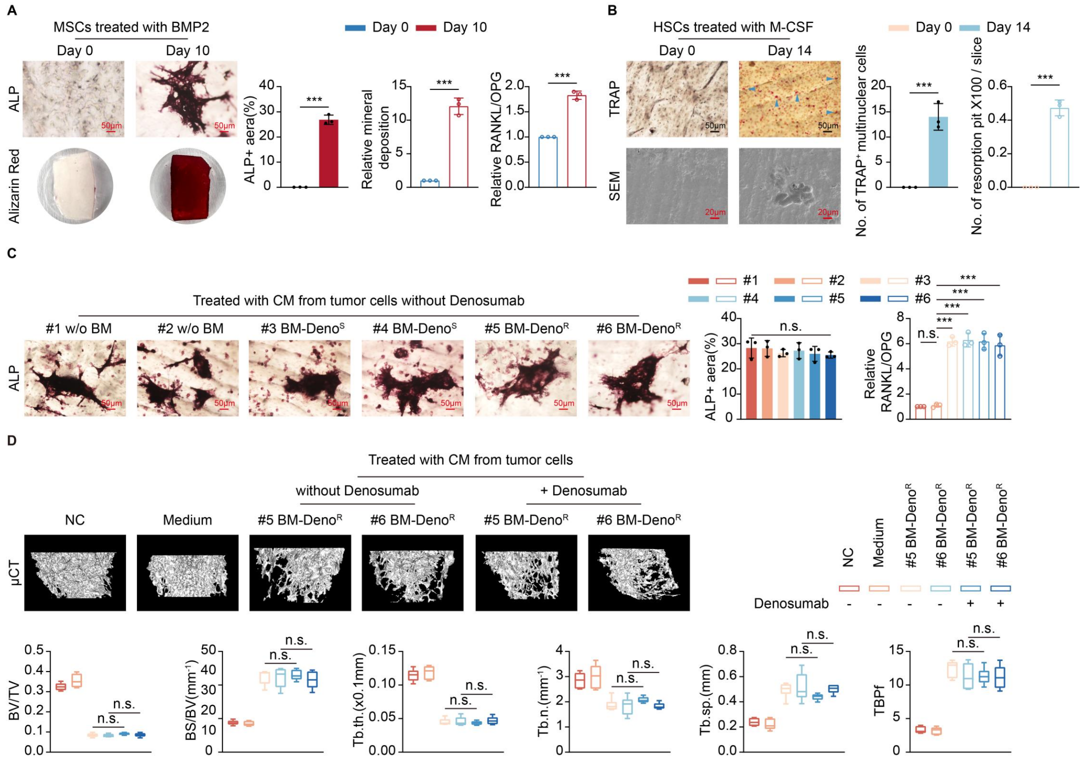

Supplementary figure 2. Differentiation of osteoblasts is not significantly affected by denosumab-resistant breast cancer cells *in vitro*.

A) Left: ALP staining (top) and Alizarin Red S mineralization (bottom) of BMP-treated mesenchymal stem cells (MSCs) at day 0 and day 10. Scale bars: 50 μm. Right: Quantification of ALP$^+$ area and mineralized matrix deposition (%). RANKL/OPG ratio in conditioned media (CM) from BMP-treated skeletal organoids.

B) Left: Representative TRAP-stained osteoclasts (scale bar: 50 μm) and SEM images of resorption pits (scale bar: 20μm). Right: TRAP+ multinucleated cell counts. Resorption pit numbers per slice.

C) Left: ALP staining of bone organoids exposed to conditioned media (CM) from primary breast cancer cells. Scale bars: 50μm. Right: Quantification of ALP$^+$ area and mineral deposition (%). RANKL /OPG ratio in CM from BMP-treated organoids.

D) Quantification of the indicated bone parameters and from representative mice (n = 6 per group). BV/TV, bone/tissue volume ratio; BS/TV, bone surface/tissue volume ratio; Tb. n, trabecular number; Tb. sp., trabecular separation; Tb. th., trabecular thickness; TBPf, trabecular bone pattern factor.

Each error bar represents the mean ± SD of 3 or more independent experiments. Significant differences were determined by one-way ANOVA with Tukey's multiple comparison test. * $p < 0.05$, ** $p < 0.01$, *** $p < 0.001$.

Supplementary figure 3

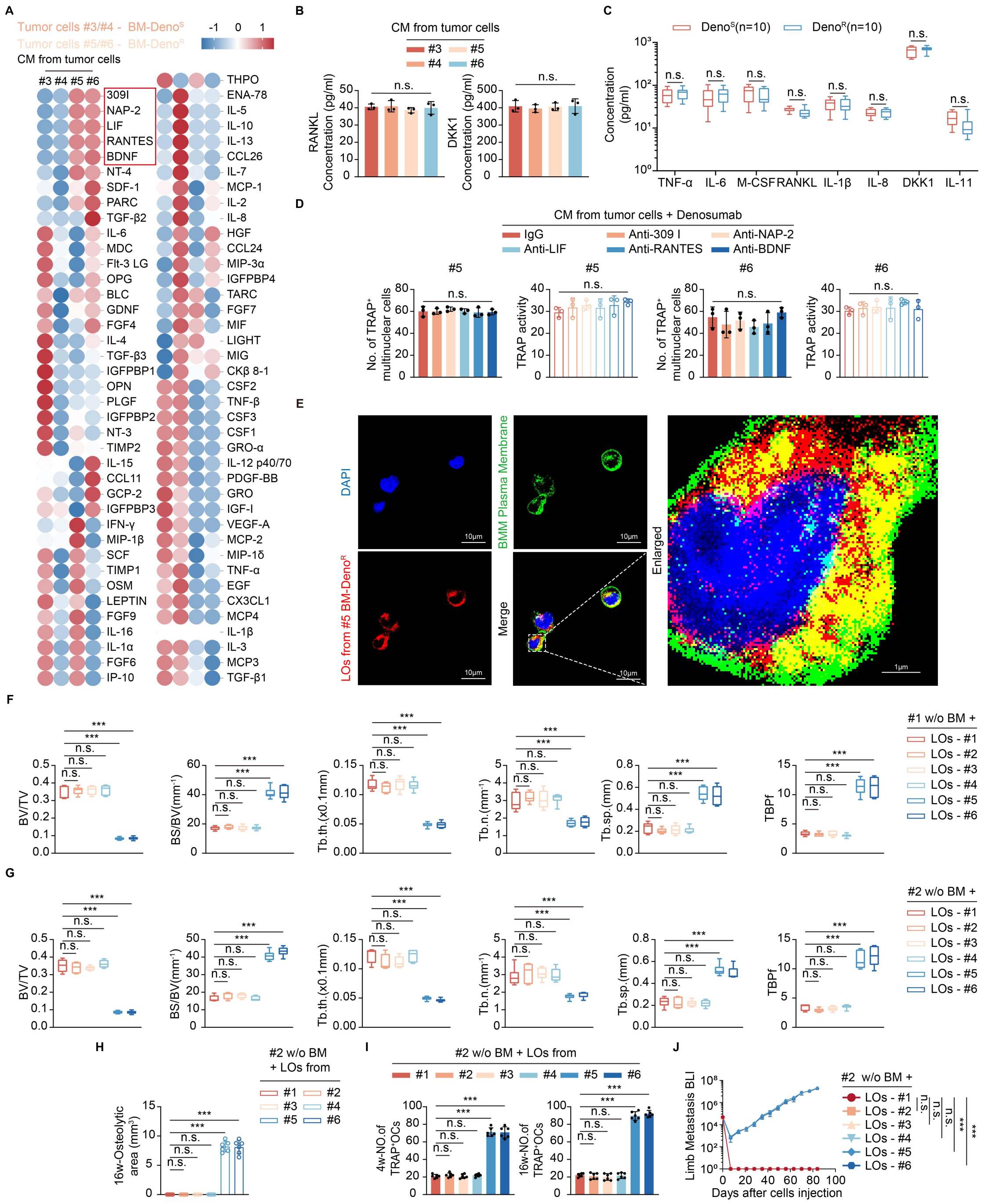

Supplementary figure 3. The uptake of LOs derived from denosumab-resistant breast cancer cells by BMMs leads to RANKL-independent osteoclast activation.

A) Heatmap of a cytokine profiling of conditioned media (CM) from primary breast cancer cells via cytokine array analysis.

B) ELISA quantification of soluble RANKL and DKK1 concentrations in CM from primary breast cancer cells.

C) Quantification of serum concentrations of classical osteoclast-activating cytokines were quantified by ELISA in denosumab-resistant and -sensitive patients (n = 10 patients/group).

D) Quantification of TRAP+ multinucleated osteoclast counts in TRAP staining of BMMs treated with CM pre-incubated with neutralizing antibodies or IgG control and TRAP enzymatic activity in corresponding groups.

E) Imaging of mouse BMMs ingesting breast cancer cell-derived LOs by confocal microscopy. BMM membranes were stained with WGA (green), and LOs were labeled with DiL (red). Scale bars: 10μm and 1μm.

F) BV/TV, bone/tissue volume ratio; BS/TV, bone surface/ tissue volume ratio; Tb. n, trabecular number; Tb. sp., trabecular separation; Tb. th., trabecular thickness; TBPf, trabecular bone pattern factor.

G) Quantification of the indicated bone parameters and number of TRAP+-osteoclasts/ALP+-osteoblasts from representative mice (n = 6 per group). BV/TV, bone/tissue volume ratio; BS/TV, bone surface/tissue volume ratio; Tb. n, trabecular number; Tb. sp., trabecular separation; Tb. th., trabecular thickness; TBPf, trabecular bone pattern factor.

H) Quantification of osteolytic area.

I) Quantification of tartrate-resistant acid phosphatase (TRAP) staining (scale bar: 50 μm) .

J) BLI images of breast cancer cells and quantification of BLI imaging of bone microenvironment colonization by non-metastatic primary breast cancer cells #2 following LO treatment.

Each error bar represents the mean ± SD of 3 or more independent experiments. Significant differences were determined by one-way ANOVA with Tukey's multiple comparison test. * $p < 0.05$, ** $p < 0.01$, *** $p < 0.001$.

Supplementary figure 4

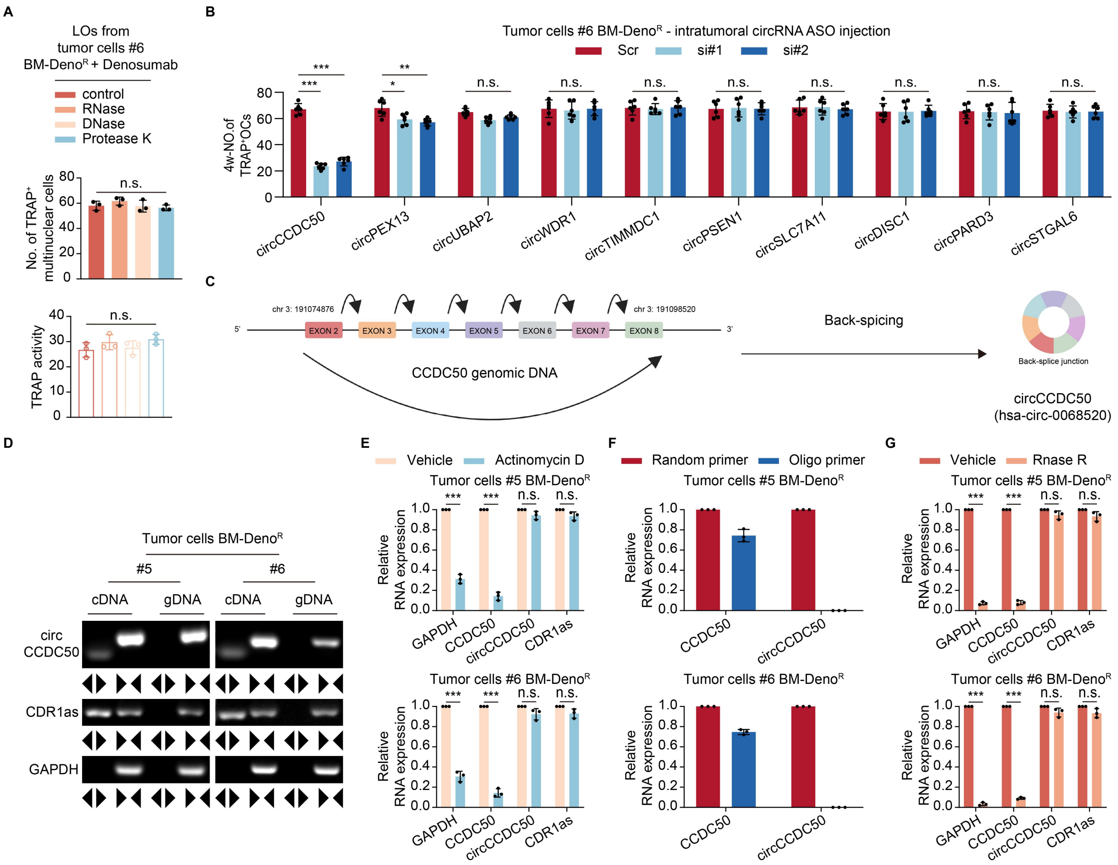

Supplementary figure 4. CircCCDC50 has a circular structure.

A) Quantification of TRAP+ multinucleated cells (upper) and TRAP activity (lower).

B) TRAP+ multinucleated osteoclast counts in bone tissues of breast cancer bone metastasis models (#5, #6) treated with circRNA-targeting siRNA or non-targeting siRNA control.

C) Schematic illustration of circCCDC50 formation via the circularization of exons 2-3-4-5-6-7-8 in CCDC50 gene. The back-splice junction sequence of circCCDC50 was validated by Sanger sequencing.

D) The existence of circCCDC50 was confirmed by RT-PCR and gel electrophoresis in primary tumor cells #5, 6 using the divergent and convergent primers, respectively. Divergent primers amplified circCCDC50 in cDNA but not genomic DNA (gDNA), along with CDR1as as a positive control, GAPDH as a negative control.

E) qRT-PCR analysis of stability of circ CCDC50 and linear CCDC50 in the Actinomycin D-treated primary tumor cells #5, 6, along with CDR1as as a positive control, GAPDH as a negative control.

F) qRT-PCR analysis of expression of circCCDC50 and linear CCDC50 using random hexamer or oligo(dT)18 primers in primary tumor cells #5, 6. GAPDH served as a loading

control.

G) qRT-PCR analysis of stability of circ CCDC50 and linear CCDC50 in the RNase R-treated primary tumor cells #5, 6, along with CDR1as as a positive control, GAPDH as a negative control.

Each error bar represents the mean ± SD of 3 or more independent experiments. Significant differences were determined by one-way ANOVA with Tukey’s multiple comparison test. * $p < 0.05$, ** $p < 0.01$, *** $p < 0.001$.

Supplementary figure 5

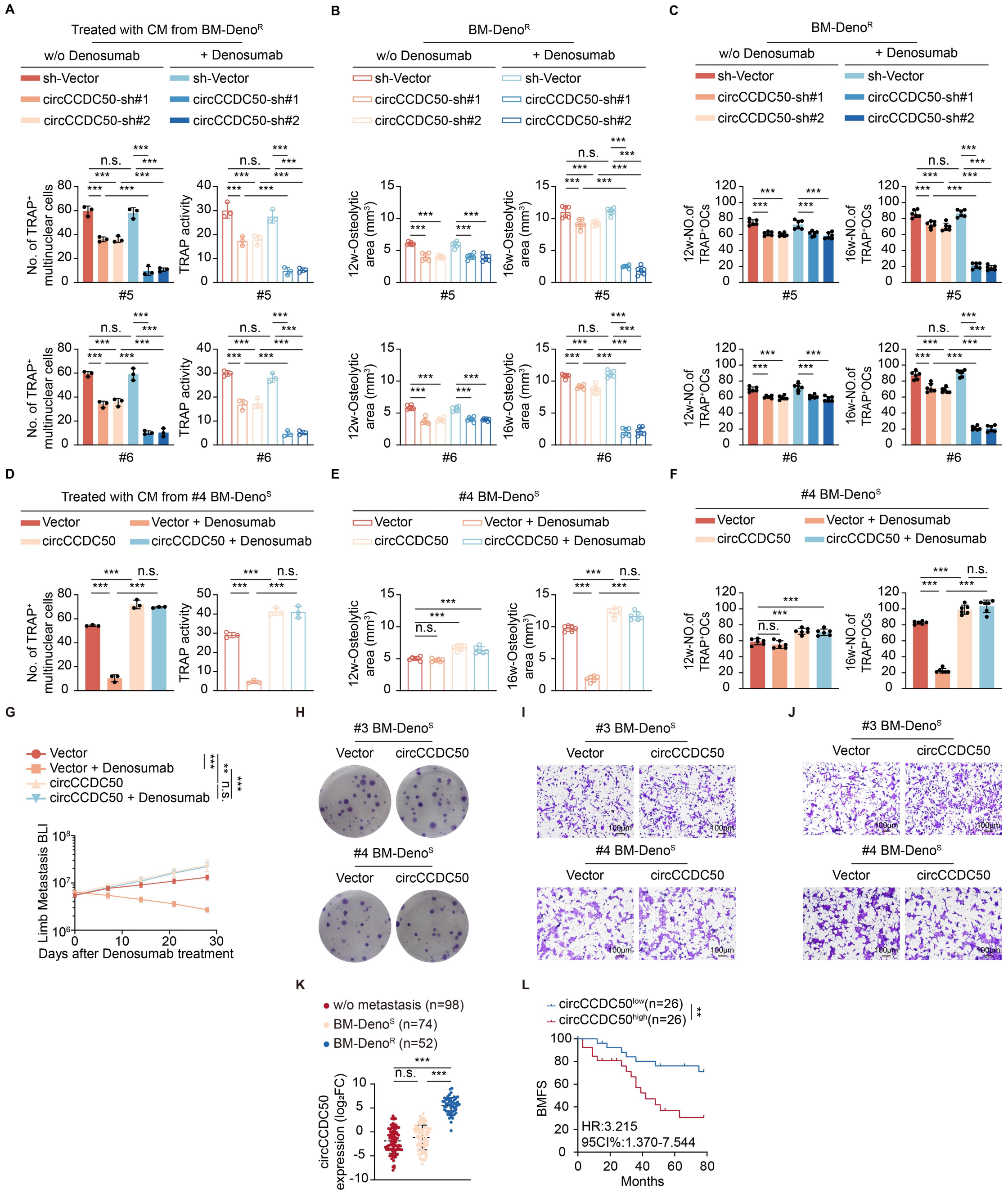

Supplementary figure 5. Overexpression of circCCDC50 promotes bone metastasis of denosumab-resistant breast cancer *in vivo*.

A) Quantification of TRAP+ multinucleated osteoclasts (OCs) in bone marrow-derived macrophages (BMMs) treated with conditioned media (CM) from primary breast cancer cells with different circCCDC50 expression, with or without Denosumab and TRAP enzymatic activity in corresponding groups.

B, C) Quantification of osteolytic area (B) and TRAP+ OCs (C) in breast cancer models implanted intobreast cancer cells #5 and #6 with different circCCDC50 expression, treated with or without Denosumab.

D) Quantification of TRAP+ multinucleated osteoclasts (OCs) in bone marrow-derived macrophages (BMMs) treated with conditioned media (CM) from breast cancer cells with different circCCDC50 expression, with or without Denosumab and TRAP enzymatic activity in corresponding groups.

E, F) Quantification of osteolytic area (E) and TRAP+ OCs (F) in breast cancer models implanted into breast cancer cells #6 with different circCCDC50 expression, treated with or without Denosumab.

G) Quantification of normalized BLI signals of mice.

H) Representative images of colony formation assays in breast cancer cells #3 and #4 with or without circCCDC50 overexpression.

I) Representative images of Transwell migration assays in breast cancer cells #3 and #4 with or without circCCDC50 overexpression.

J) Representative images of Matrigel-coated Transwell invasion assays in breast cancer cells #3 and #4 with or without circCCDC50 overexpression.

K) Analysis of plasma circCCDC50 levels in three patient cohorts: non-metastatic breast cancer (n=98); Denosumab-sensitive bone metastasis (n=74); and Denosumab-resistant bone metastasis (n=51).

L) Survival analysis of plasma circCCDC50 levels and patient BMFS.

Each error bar represents the mean ± SD of 3 or more independent experiments. Significant differences were determined by one-way ANOVA with Tukey's multiple comparison test. * $p < 0.05$, ** $p < 0.01$, *** $p < 0.001$.

Supplementary figure 6

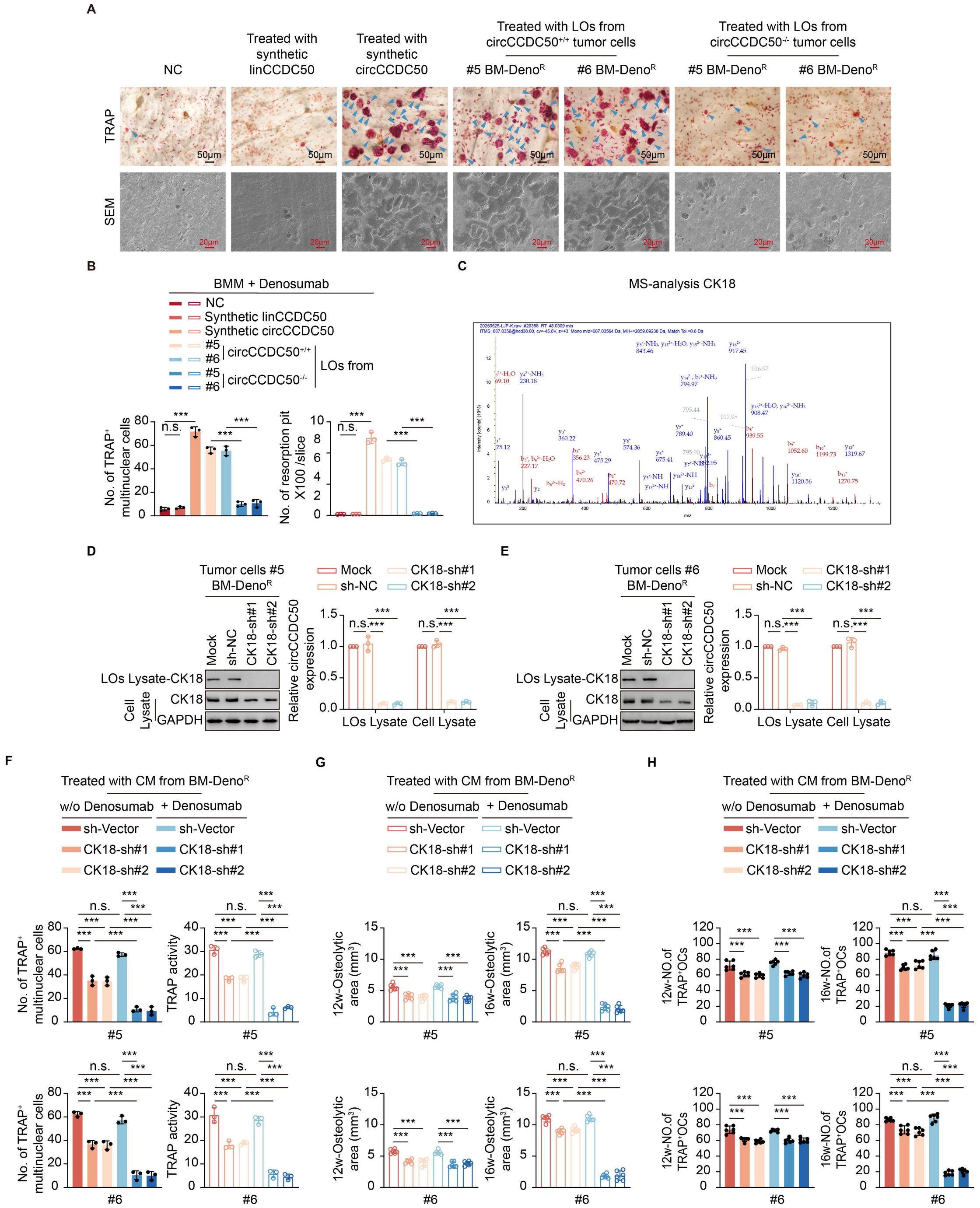

Supplementary figure 6. circCCDC50 knockout leads to a significant impairment in osteoclast activation by denosumab-resistant breast cancer cells *in vitro*.

A) Representative TRAP-stained images (scale bar: 50μm) and SEM micrographs (scale bar: 20μm) of skeletal organoid models treated with Denosumab in combination with either synthetic linear CCDC50 mRNA, circCCDC50, or conditioned medium.

B) Quantification of TRAP^{+} multinucleated cells (left) and resorption pits per section (right) in treated skeletal organoid models.

C) Proteins co-precipitated with circCCDC50 were identified via ChIRP-MS. CK18 exhibited

high binding affinity.

D&E) qRT-PCR detection of circCCDC50 levels in cell lysates and LO lysates of primary tumor cells #5(D) and #6(E).

F) Quantification of TRAP+ multinucleated osteoclasts (OCs) in bone marrow-derived macrophages (BMMs) treated with conditioned media (CM) from breast cancer cells with different CK18 expression, with or without Denosumab and TRAP enzymatic activity in corresponding groups.

G&H) Quantification of osteolytic area (G) and TRAP+ OCs (H) in breast cancer models implanted into breast cancer cells #5 or #6 with different CK18 expression, treated with or without Denosumab.

Each error bar represents the mean ± SD of 3 or more independent experiments. Significant differences were determined by one-way ANOVA with Tukey's multiple comparison test. * $p < 0.05$, ** $p < 0.01$, *** $p < 0.001$.

Supplementary figure 7

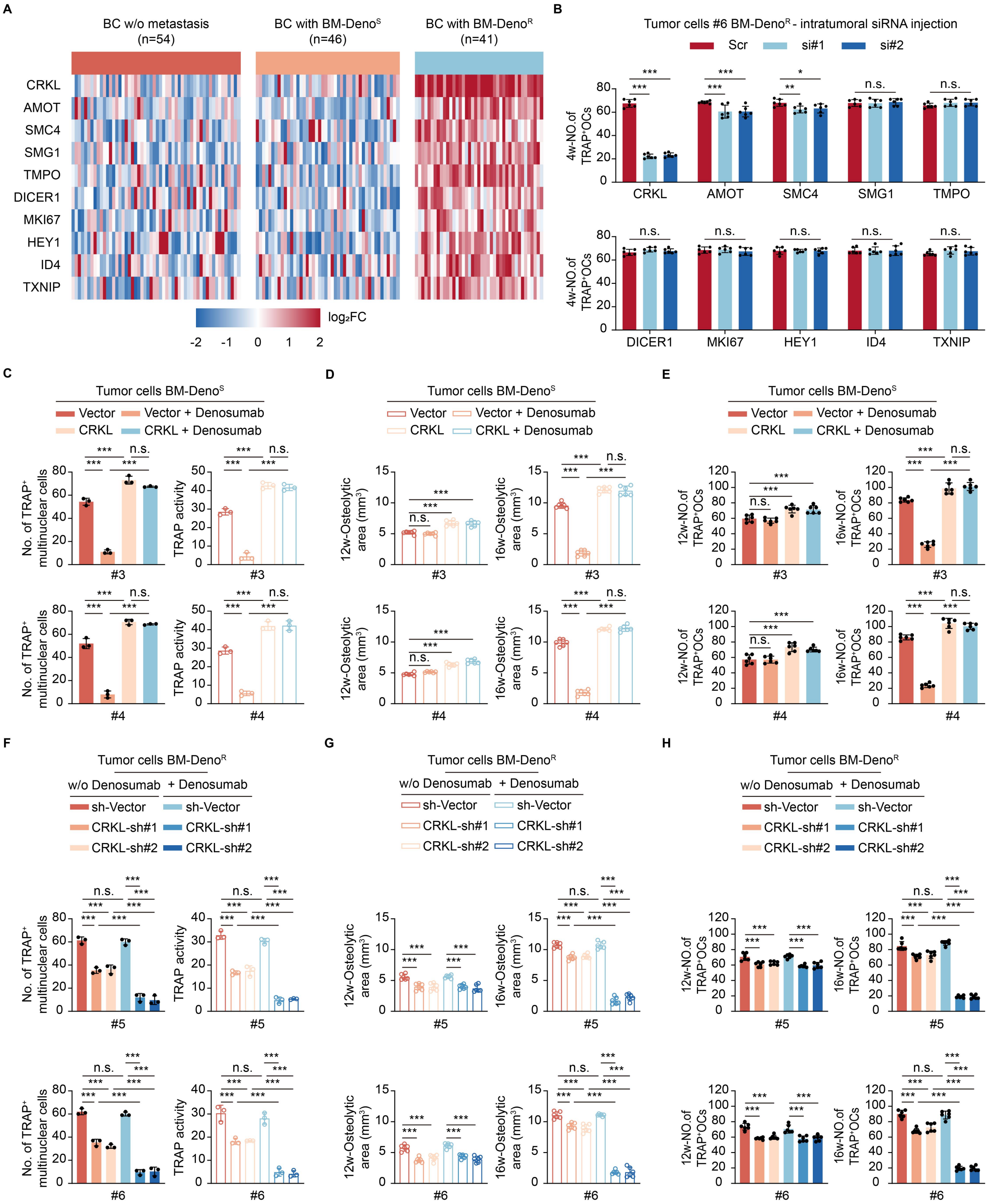

Supplementary figure 7. Overexpression of CRKL promotes bone metastasis of denosumab-resistant breast cancer *in vitro* and *vivo*.

A) Heatmap of RT-qPCR validation results for differentially expressed genes (DEGs) identified through transcriptomic sequencing and pathway enrichment analysis.

B) TRAP+ multinucleated osteoclast counts in bone tissues of breast cancer models #6 treated with dysregulated mRNAs -targeting siRNA or non-targeting siRNA control (n = 6 mice/group).

C) Quantification of TRAP+ multinucleated osteoclasts (OCs) in bone marrow-derived

macrophages (BMMs) treated with conditioned media (CM) from primary breast cancer cells with different CRKL expression, with or without Denosumab and TRAP enzymatic activity in corresponding groups.

D, E) Quantification of osteolytic area (D) and TRAP+ OCs (E) in breast cancer models implanted into breast cancer cells #5 and #6 with different CRKL expression, treated with or without Denosumab.

F) Quantification of TRAP+ multinucleated osteoclasts (OCs) in bone marrow-derived macrophages (BMMs) treated with conditioned media (CM) from primary breast cancer cells with different CRKL expression, with or without Denosumab and TRAP enzymatic activity in corresponding groups.

G, H) Quantification of osteolytic area (G) and TRAP+ OCs (H) in breast cancer models implanted into breast cancer cells #5 and #6 with different CRKL expression, treated with or without Denosumab.

Each error bar represents the mean ± SD of 3 or more independent experiments. Significant differences were determined by one-way ANOVA with Tukey's multiple comparison test. * $p < 0.05$, ** $p < 0.01$, *** $p < 0.001$.

Supplementary figure 8

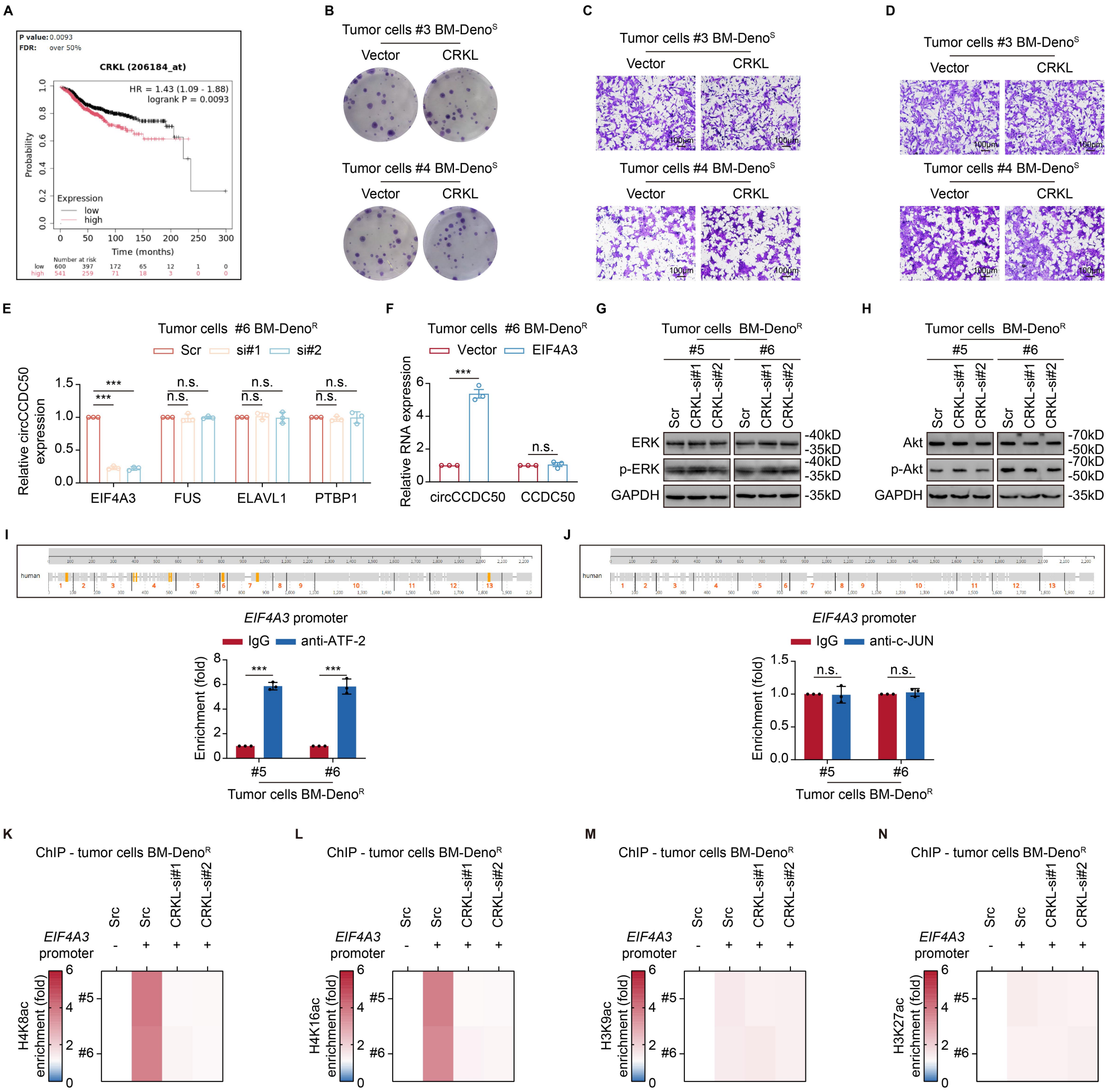

Supplementary figure 8. Binding of ATF-2 to the EIF4A3 promoter induces histone H3 and H4 acetylation.

A) Survival analysis of CRKL expression and patient prognosis based on the Kaplan–Meier plotter database.

B) Representative images of colony formation assays in breast cancer cells #3 and #4 with or without CRKL overexpression.

C) Representative images of Transwell migration assays in breast cancer cells #3 and #4 with or without CRKL overexpression.

D) Representative images of Matrigel-coated Transwell invasion assays in breast cancer cells #3 and #4 with or without CRKL overexpression.

E) Real-time PCR analysis of circCCDC50 expression in primary tumor cells #6. GAPDH served as a loading control.

F) Real-time PCR analysis of circCCDC50 and CCDC50 expression in

EIF4A3-overexpressing and control tumor cells #6. GAPDH served as a loading control.

G) Immunoblots of phosphorylated ERK (p-ERK) and total ERK (t-ERK) in tumor cells transfected with CRKL-targeting siRNA (si-CRKL) or scrambled siRNA control (si-Ctrl). GAPDH served as a loading control.

H) Immunoblots of phosphorylated Akt (p-Akt) and total Akt (t-Akt) in tumor cells transfected with CRKL-targeting siRNA (si-CRKL) or scrambled siRNA control (si-Ctrl). GAPDH served as a loading control.

I) Bioinformatically predicted ATF-2 binding sites on the NFATc1 promoter and validated by ChIP-qPCR.

J) Bioinformatically predicted ATF-2:c-JUN heterodimer binding sites on the NFATc1 promoter and validated by ChIP-qPCR.

K-N) H4K8ac (K), H4K16ac (L), H3K9ac (M), and H3K27ac (N) enrichment at the EIF4A3 promoter region was elevated in resistant cells by ChIP-qPCR. EIF4A3 promoter (+) indicates the H4K8ac, H4K16ac, H3K9ac, and H3K27ac enrichment at the EIF4A3 promoter region; EIF4A3 promoter (-) indicates the enrichment at a negative control region.

Each error bar represents the mean ± SD of 3 independent experiments. Significant differences were determined by one-way ANOVA with Tukey’s multiple comparison test. * $p < 0.05$, ** $p < 0.01$, *** $p < 0.001$.

Supplementary figure 9

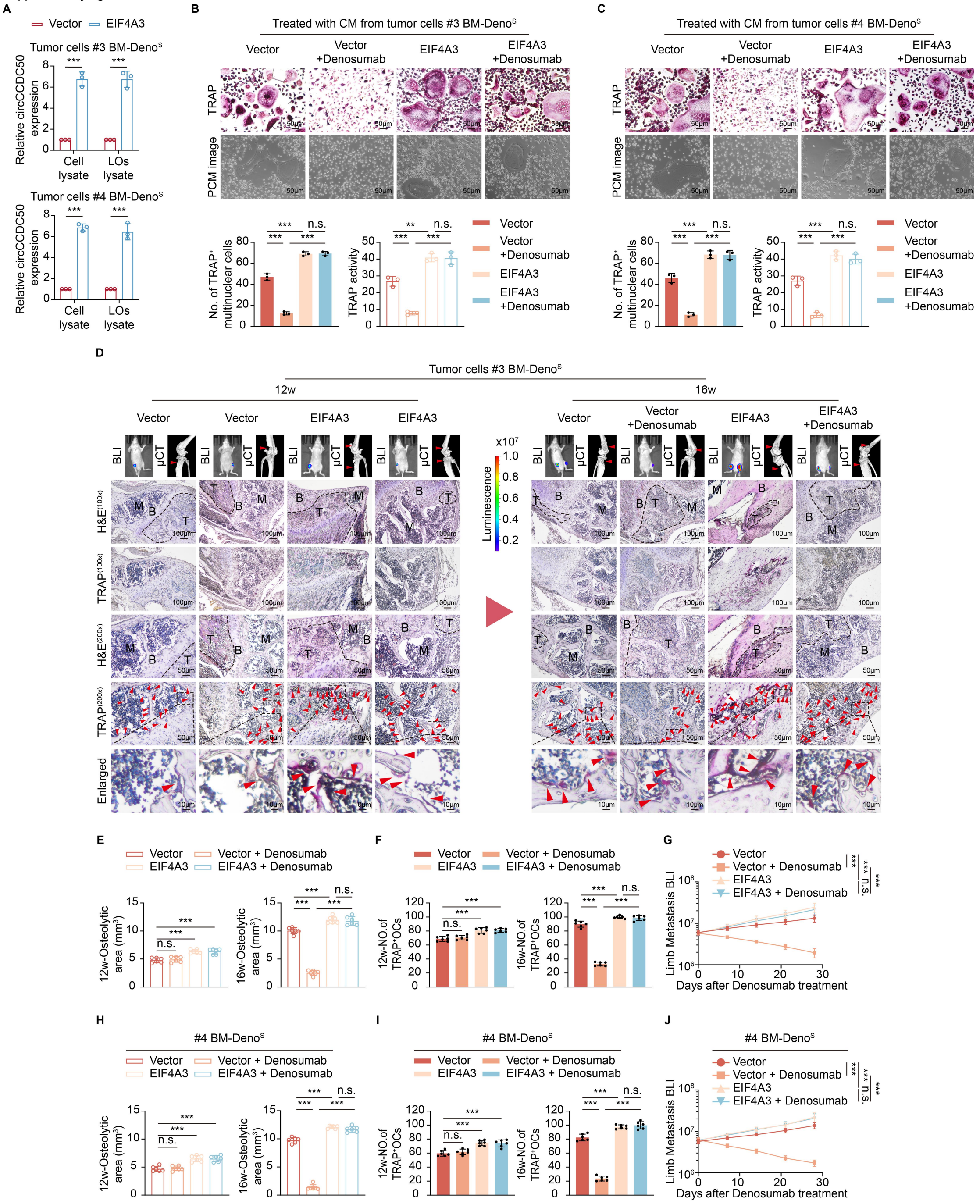

Supplementary figure 9. Overexpression of EIFA43 promotes bone metastasis of denosumab-resistant breast cancer *in vitro* and *vivo*.

A) qPCR analysis of EIF4A3 mRNA expression in breast cancer cells with or without EIF4A3 overexpression and LOs derived from them.

B) Quantification of TRAP+ multinucleated osteoclasts (OCs) in bone marrow-derived macrophages (BMMs) treated with conditioned media (CM) from primary breast cancer cells #3 BM-Deno^S with different EIF4A3 expression, with or without Denosumab and TRAP enzymatic activity in corresponding groups.

C) Quantification of TRAP+ multinucleated osteoclasts (OCs) in bone marrow-derived

macrophages (BMMs) treated with conditioned media (CM) from primary breast cancer cells #4 BM-Deno^{S} with different EIF4A3 expression, with or without Denosumab and TRAP enzymatic activity in corresponding groups.

D) BLI images and μCT scans of bone tissues from breast cancer models, Representative images of hematoxylin and eosin (H&E) staining, tartrate-resistant acid phosphatase (TRAP) staining. Scale bar: 100 μm (100x), 50 μm (200x) and 10 μm (Enlarged).

E) Quantitative analysis of osteolytic lesion area via μCT in mice (n = 6 per group).

F) Quantitative analysis of TRAP+ multinucleated cell counts per field in mice (n = 6 per group).

G) Quantification of normalized BLI signals of mice (n = 6 per group).

H) Quantitative analysis of osteolytic lesion area via μCT in mice (n = 6 per group).

I) Quantitative analysis of TRAP+ multinucleated cell counts per field in mice (n = 6 per group).

J) Quantification of normalized BLI signals of mice (n = 6 per group).

Each error bar represents the mean ± SD of 3 or more independent experiments. Significant differences were determined by one-way ANOVA with Tukey's multiple comparison test. * $p < 0.05$, ** $p < 0.01$, *** $p < 0.001$.

Supplementary figure 10

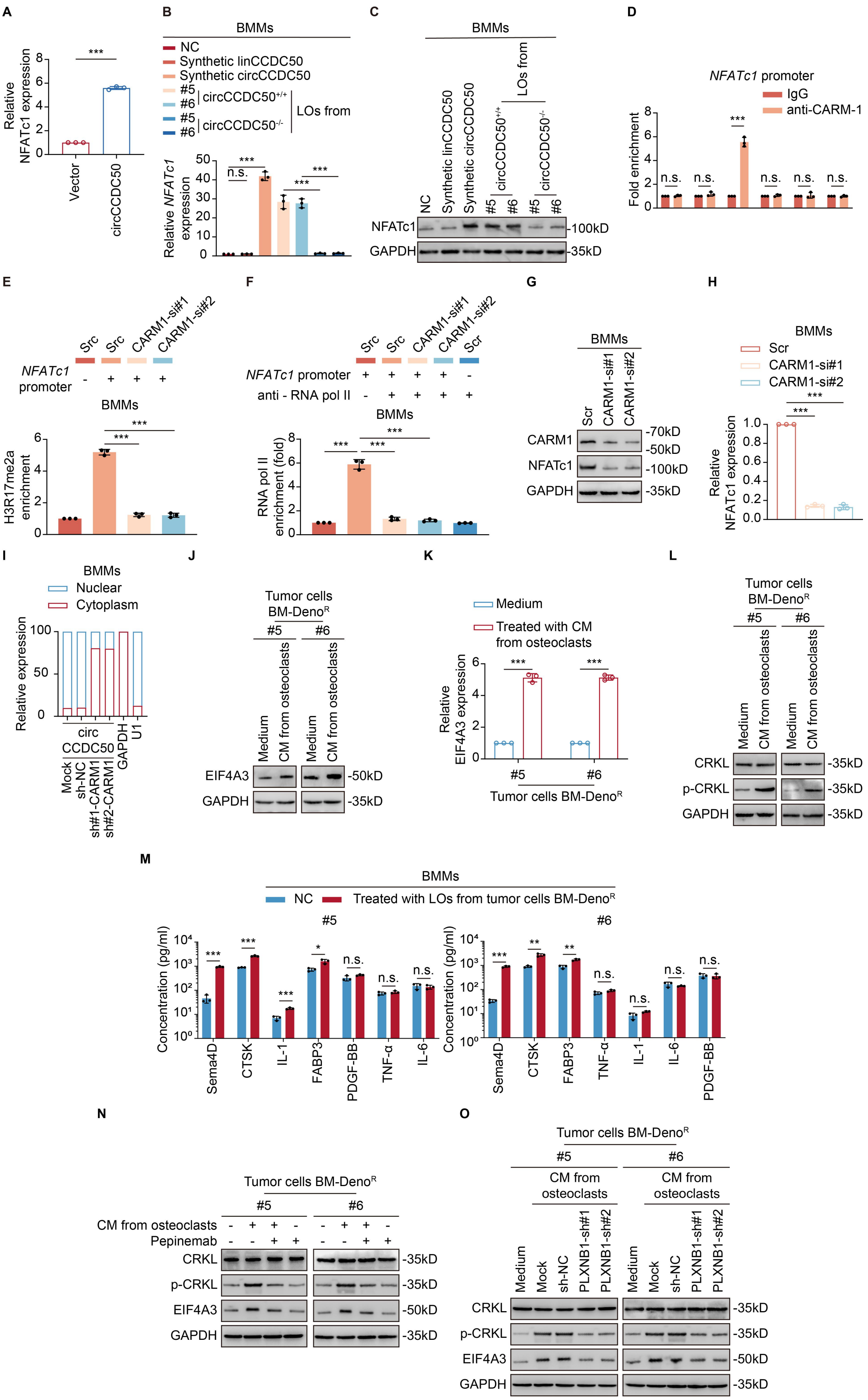

Supplementary figure 10. SEMA4D secreted by osteoclasts upregulates EIF4A3

expression in denosumab-resistant breast cancer cells.

A) qRT-PCR analysis of NFATc1 expression in BMMs with or without circCCDC50 overexpression.

B) qPCR analysis of NFATc1 mRNA expression in mice BMMs treated with Denosumab in combination with synthetic linear CCDC50 mRNA, circCCDC50, or conditioned medium.

C) Western blot analysis of NFATc1 protein expression in mice BMMs treated with Denosumab in combination with synthetic linear CCDC50 mRNA, circCCDC50, or conditioned medium.

D) ChIP-qPCR validation of CARM1 binding to the NFATc1 promoter.

E) H3R17me2a enrichment at the NFATc1 promoter region was elevated in BMMs with or without CARM1 knockdown by ChIP-qPCR. NFATc1 promoter (+) indicates the H3R17me2a enrichment at the NFATc1 promoter region; NFATc1 promoter (-) indicates the enrichment at a negative control region.

F) RNA pol II occupancy at the NFATc1 promoter was elevated in BMMs with or without CARM1 knockdown by ChIP-qPCR. NFATc1 promoter (+) indicates the RNA pol II enrichment at the NFATc1 promoter region; NFATc1 promoter (-) indicates the RNA pol II enrichment at a negative control region. anti-RNA pol II (+) indicates ChIP with an anti-RNA polymerase II antibody; anti-RNA pol II (-) indicates ChIP with a control IgG antibody.

G) Western-blot analysis of NFATc1 protein in CARM1-knockdown BMMs

H) qRT-PCR analysis of NFATc1 mRNA in CARM1-knockdown BMMs.

I) Nuclear-cytoplasmic fractionation assay to assess circCCDC50 distribution in CARM1-knockdown BMMs.

J) Immunoprecipitation analysis of EIF4A3 expression in primary breast cancer cells (#5, #6) treated with osteoclast-conditioned medium (CM).

K) qRT-PCR analysis of EIF4A3 mRNA in CM-treated breast cancer cells.

L) Immunoprecipitation analysis of CRKL phosphorylation in CM-treated breast cancer cells.

M) ELISA quantification of cytokine secretion in osteoclasts treated with LOs from breast cancer cells (#5, #6).

N) Western-blot analysis of CRKL phosphorylation and EIF4A3 levels in breast cancer cells treated with CM ± Pepinemab (SEMA4D inhibitor).

O) Western-blot analysis of CRKL phosphorylation and EIF4A3 levels in

PLXNB1-knockdown breast cancer cells treated with CM.

Each error bar represents the mean ± SD of 3 independent experiments. Significant differences were determined by one-way ANOVA with Tukey's multiple comparison test. * $p < 0.05$, ** $p < 0.01$, *** $p < 0.001$.

Supplementary figure 11

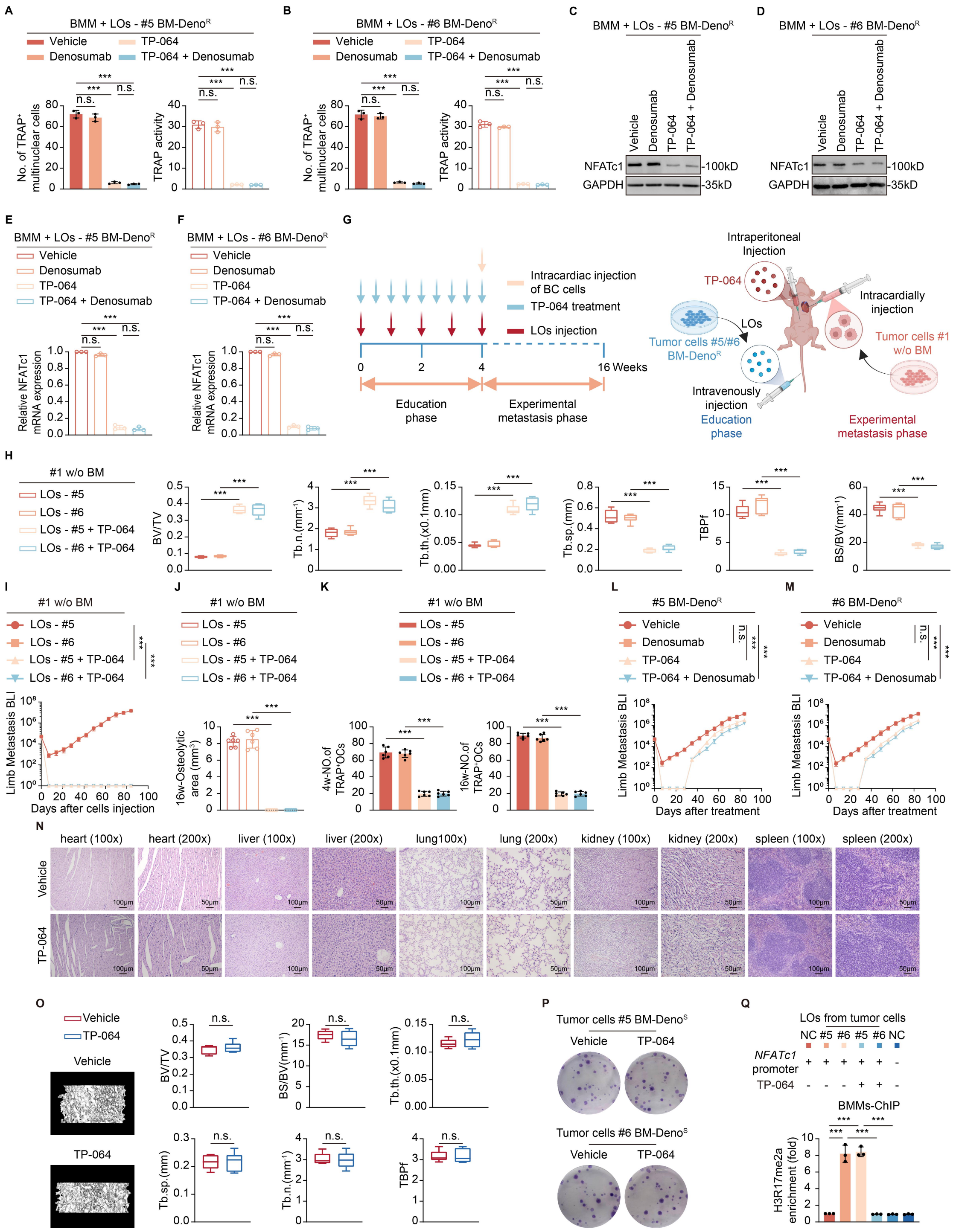

Supplementary figure 11. Administration of TP-064 inhibits the colonization of

denosumab-resistant breast cancer cells in the bone microenvironment *in vivo*.

A, B) TRAP+ multinucleated cell counts in BMMs exposed to CM from resistant cells #5 (A) and #6 (B) treated with denosumab, TP-064, or combination therapy. TRAP enzymatic activity (nmol pNP/min/mg protein) in corresponding groups.

C, D) NFATc1 protein levels were evaluated in denosumab-resistant primary breast cancer cell lines #5 (C), #6 (D), treated with or without TP-064. GAPDH served as a loading control.

E, F) NFATc1 relative mRNA expression were evaluated in denosumab-resistant primary breast cancer cell lines #5 (E), #6 (F), treated with or without TP-064 by qRT-PCR.

G) Schematic overview of the pre-metastatic niche education and experimental metastasis mouse models.

H) Quantification of the indicated bone parameters and from representative mice (n = 6 per group). BV/TV, bone/tissue volume ratio; BS/TV, bone surface/tissue volume ratio; Tb. n, trabecular number; Tb. sp., trabecular separation; Tb. th., trabecular thickness; TBPf, trabecular bone pattern factor.

I) Quantification of normalized BLI signals in animal models with different treatments.

J) µCT-based analysis of osteolytic lesion area in bone tissues of breast cancer bone metastasis mouse models models treated with indicated therapies.

K) Quantification of TRAP+ multinucleated osteoclasts per field in bone tissues of breast cancer bone metastasis mouse models treated with indicated therapies.

L) Quantification of normalized BLI signals in #5 animal models with different treatments.

M) Quantification of normalized BLI signals in #6 animal models with different treatments.

O) Left: Representative µCT images of trabecular bone from humanized NCG-hairless mice treated with or without TP-064. Right: Quantification of the indicated bone parameters and from representative mice (n = 6 per group). BV/TV, bone/tissue volume ratio; BS/TV, bone surface/tissue volume ratio; Tb. n, trabecular number; Tb. sp., trabecular separation; Tb. th., trabecular thickness; TBPf, trabecular bone pattern factor.

P) Colony formation assay of Denosumab-resistant ER-positive breast cancer cells treated with or without 0.1 µM TP-064.

Q) H3R17me2a enrichment at the NFATc1 promoter region was elevated in BMMs derived from NCG-hairless mice following treatment with or without LOs and with or without TP-064 by ChIP-qPCR. NFATc1 promoter (+) indicates the H3R17me2a enrichment at the NFATc1 promoter region; NFATc1 promoter (-) indicates the enrichment at a negative control

region. TP-064 (+) indicates NCG-hairless mice treated with TP-064; TP-064 (-) indicates NCG-hairless mice treated without TP-064.

Each error bar represents the mean ± SD of 3 or more independent experiments. Significant differences were determined by one-way ANOVA with Tukey's multiple comparison test. * $p < 0.05$, ** $p < 0.01$, *** $p < 0.001$.

Supplementary table

Supplementary Table 1. Baseline and characteristics of patients.

Patients number	1	2	3	4	5	6
Age, years	56	61	59	57	63	61
ER receptor status	+	+	+	+	+	+
PR receptor status	+	+	+	+	+	+
HER2 status	IHC 1+	IHC 0	IHC 1+	IHC 1+	IHC 1+	IHC 0
Ki67 expression	10%	15%	5%	15%	10%	10%
Molecular subtype	Luminal A	Luminal A	Luminal A	Luminal A	Luminal A	Luminal A
Clinically TNM stage	IV	IV	IV	IV	IV	IV
Bone metastasis	-	-	+	+	+	+
Visceral metastasis	-	-	-	-	-	-

HR: hormone receptor. PR: Progesterone Receptor. HER2: human epidermal growth factor receptor 2. IHC: immunochemistry.
