# Supplementary material for: Targeting RANKL-independent osteoclastogenesis overcomes denosumab resistance in models of ER+ breast cancer bone metastasis
Source: J Clin Invest. 2026 May 15;136(10):e199285. doi: 10.1172/JCI199285 (PMC13178652; doi:10.1172/JCI199285)

Full unedited blot for Figure 3A

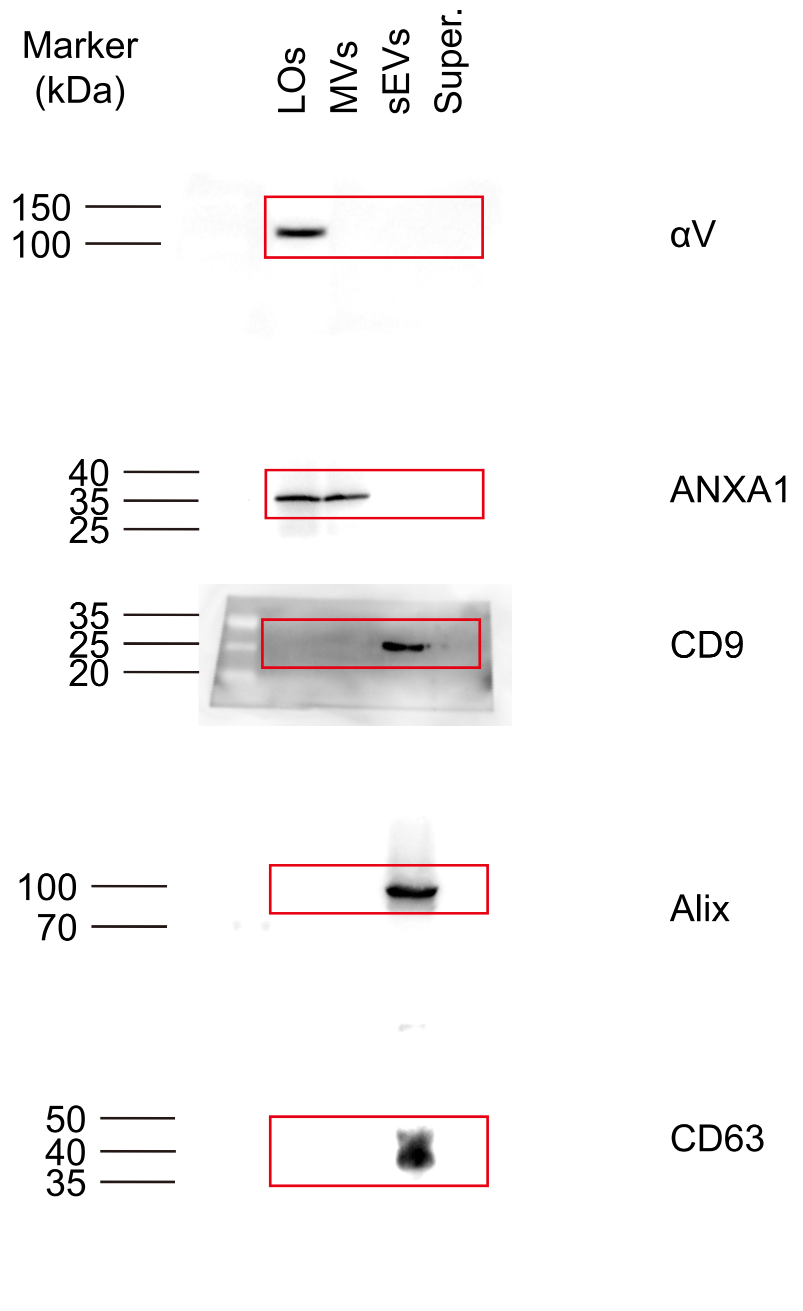

Full unedited blot for Figure 8D

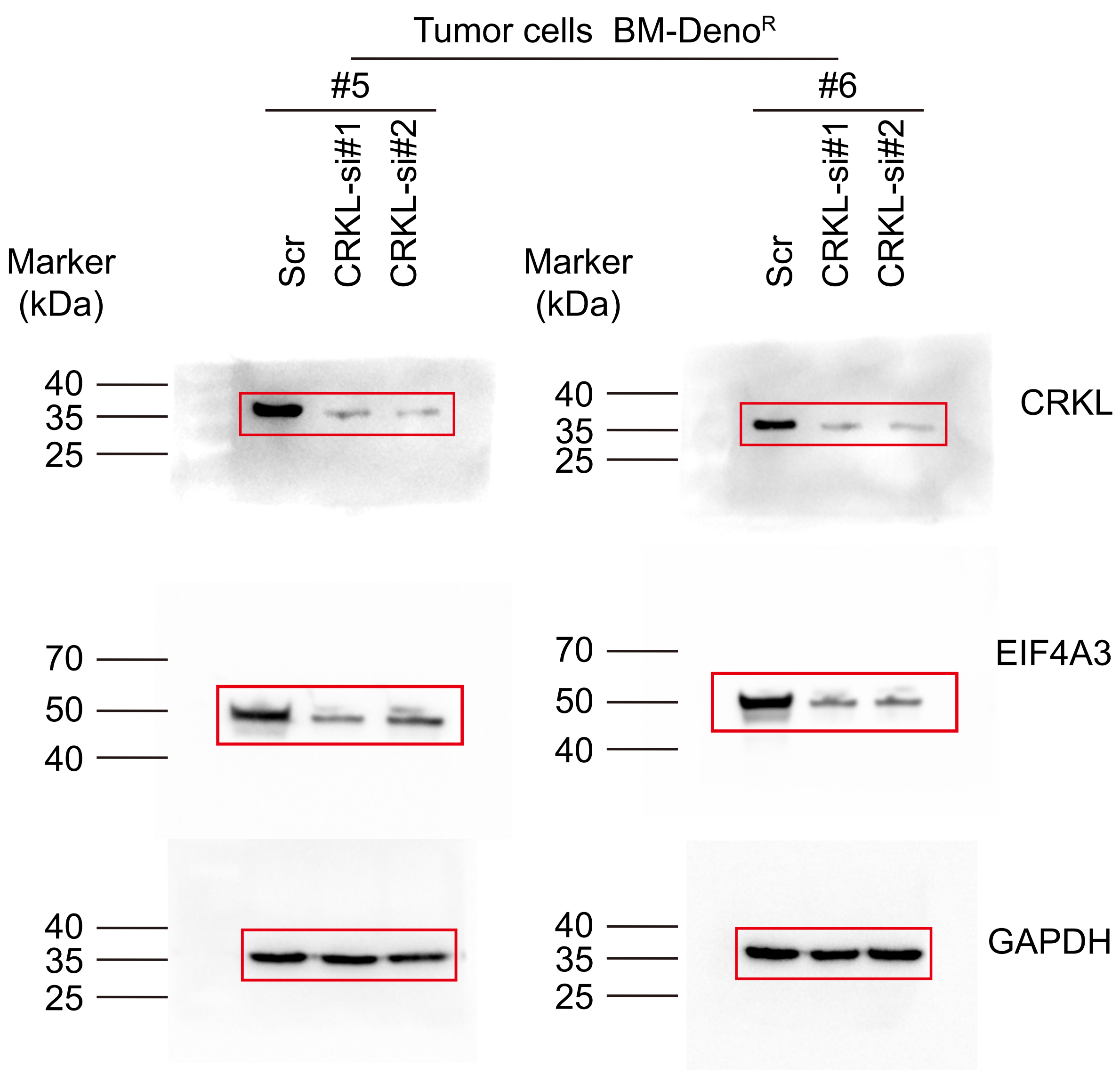

Full unedited blot for Figure 8E

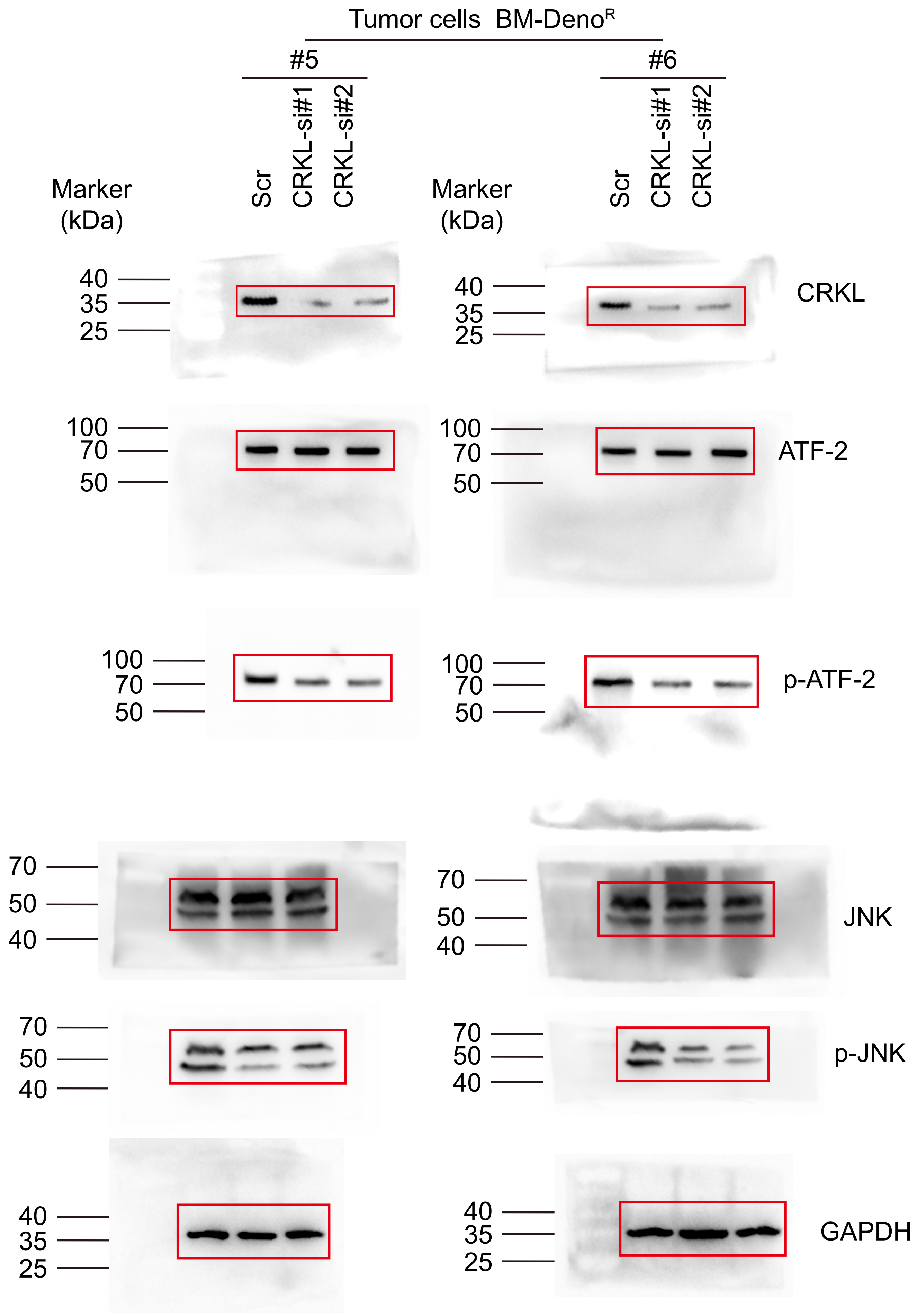

Full unedited blot for Figure 8F

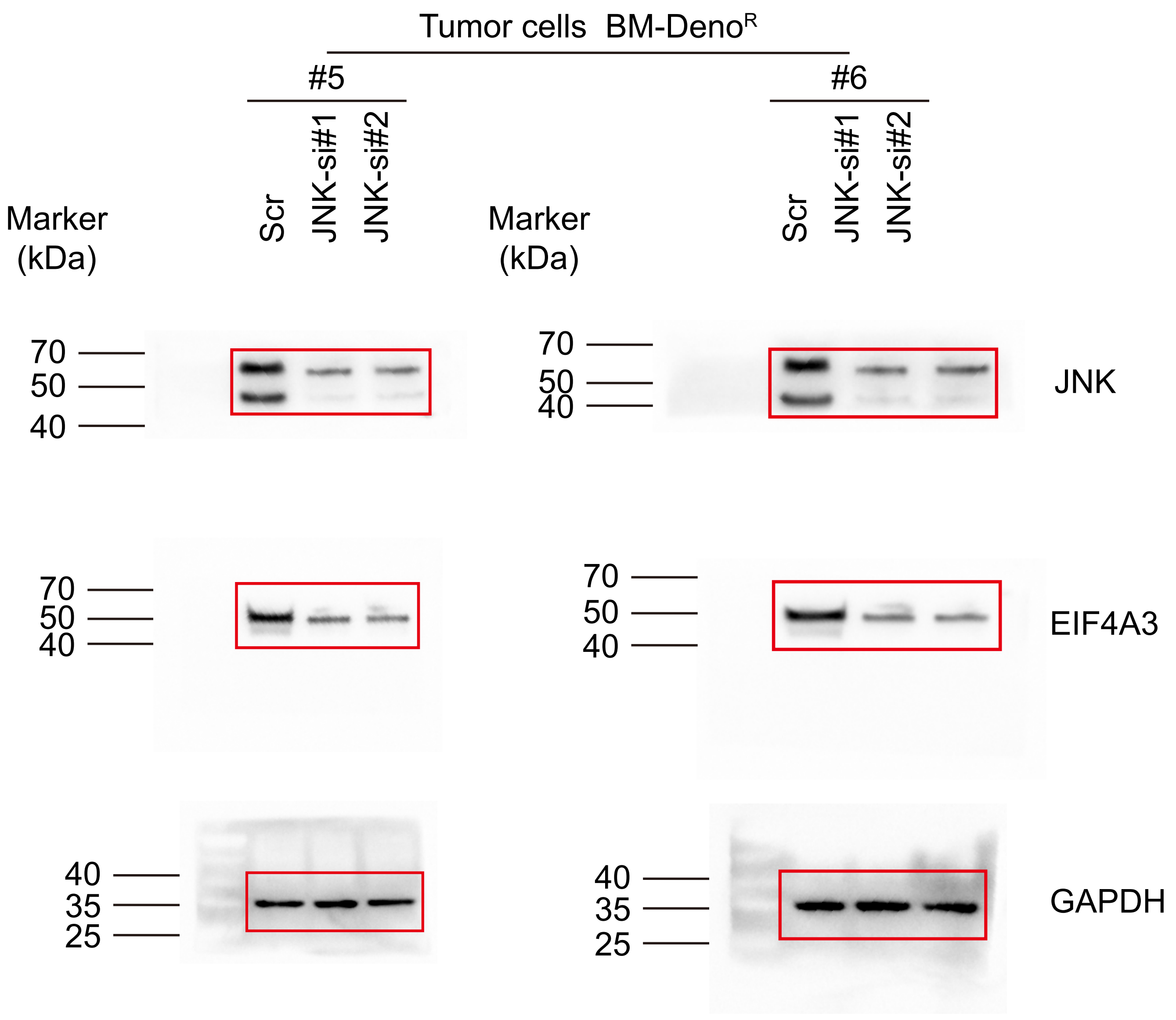

Full unedited blot for Figure 8G

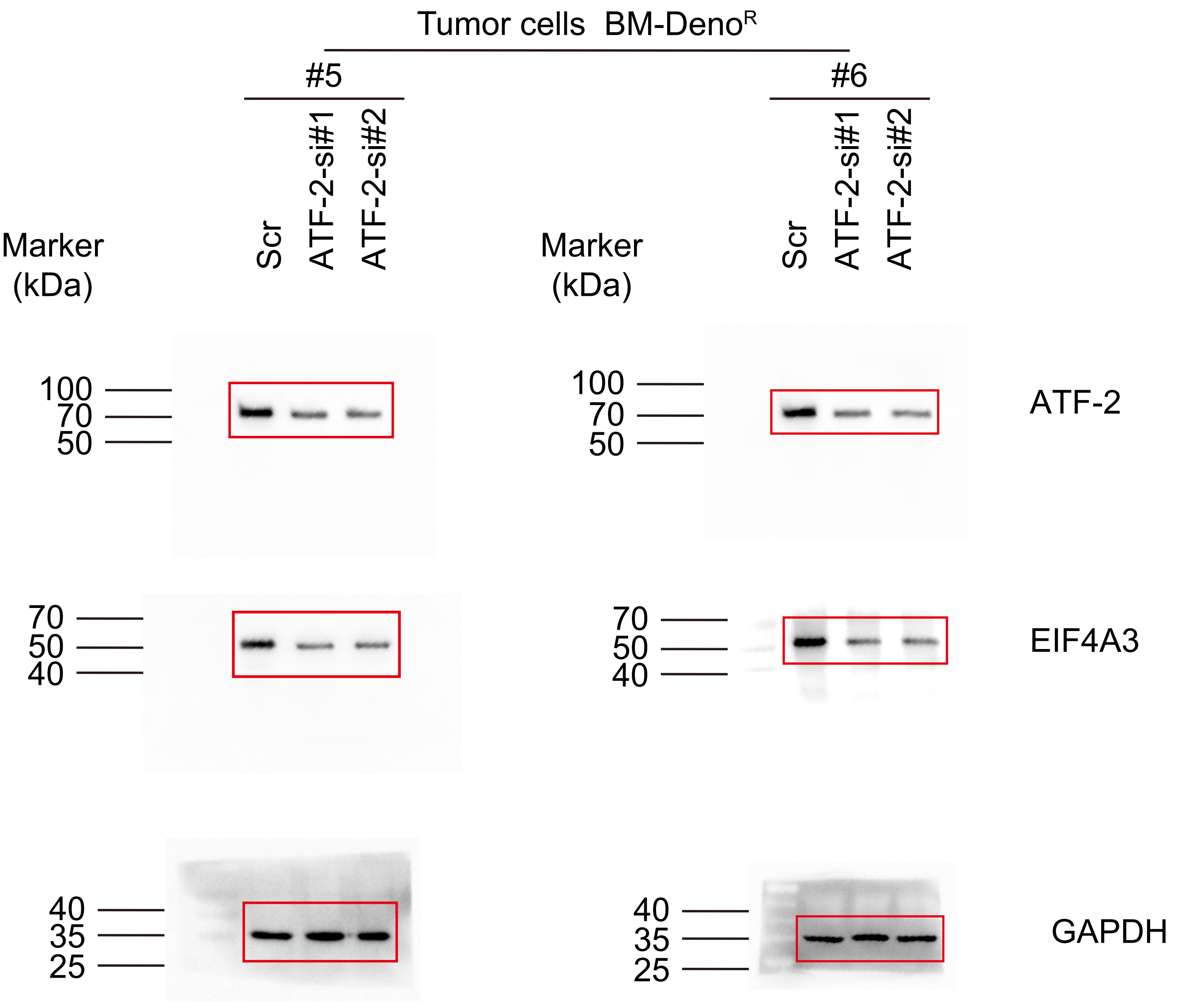

Full unedited blot for Figure 9B

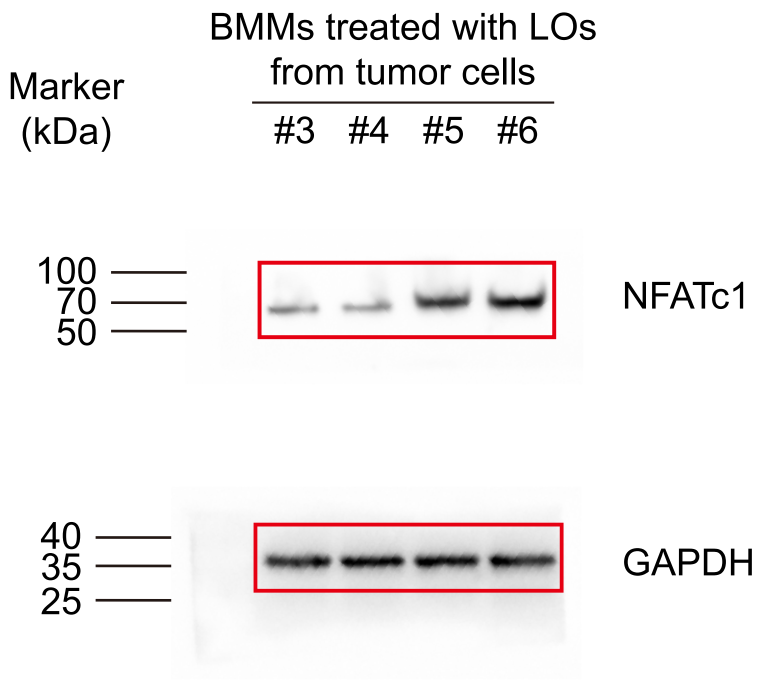

Full unedited blot for Figure 9H

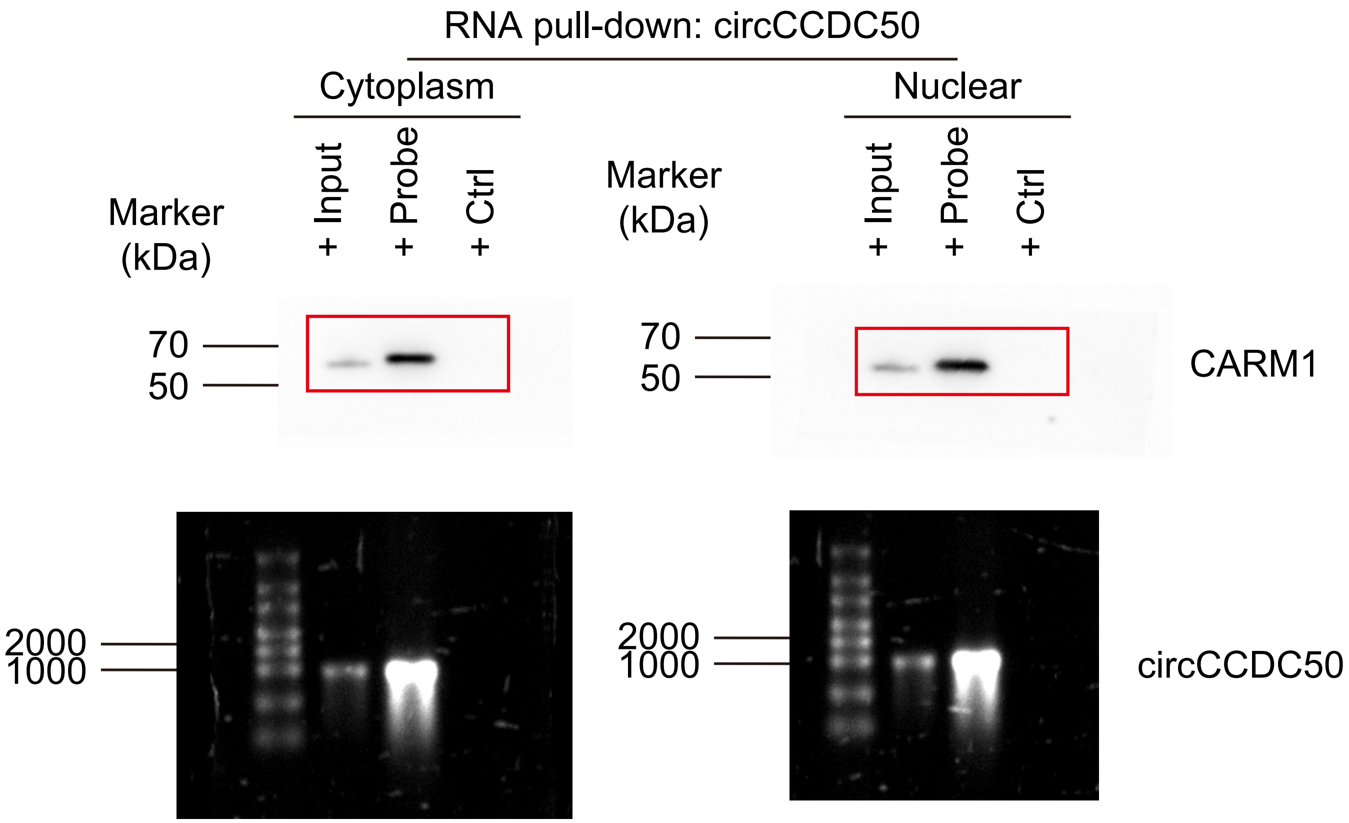

Full unedited blot for Figure 10D

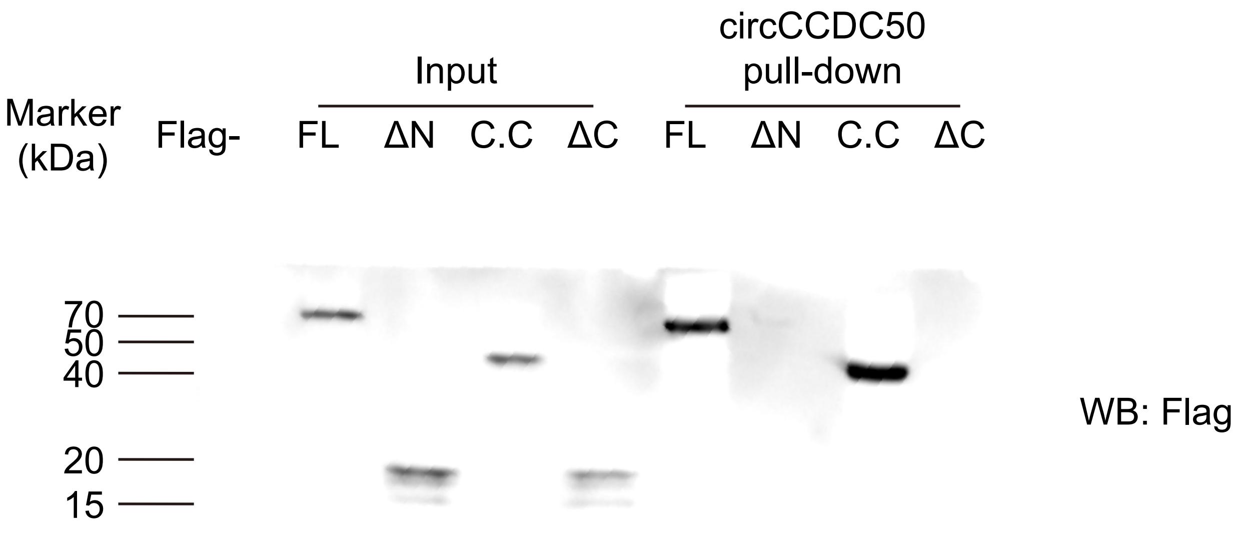

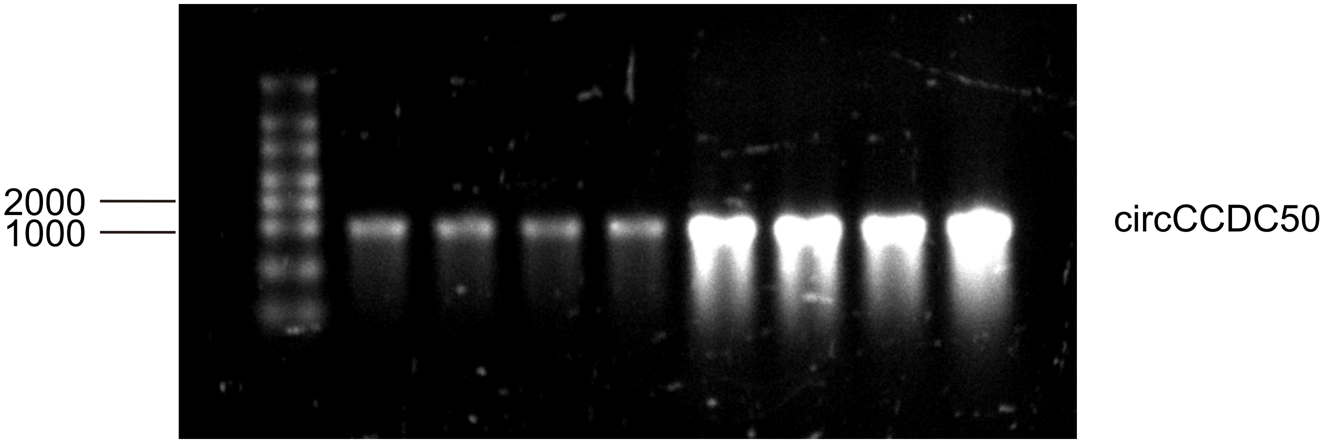

Full unedited blot for Supplementary Figure 4D

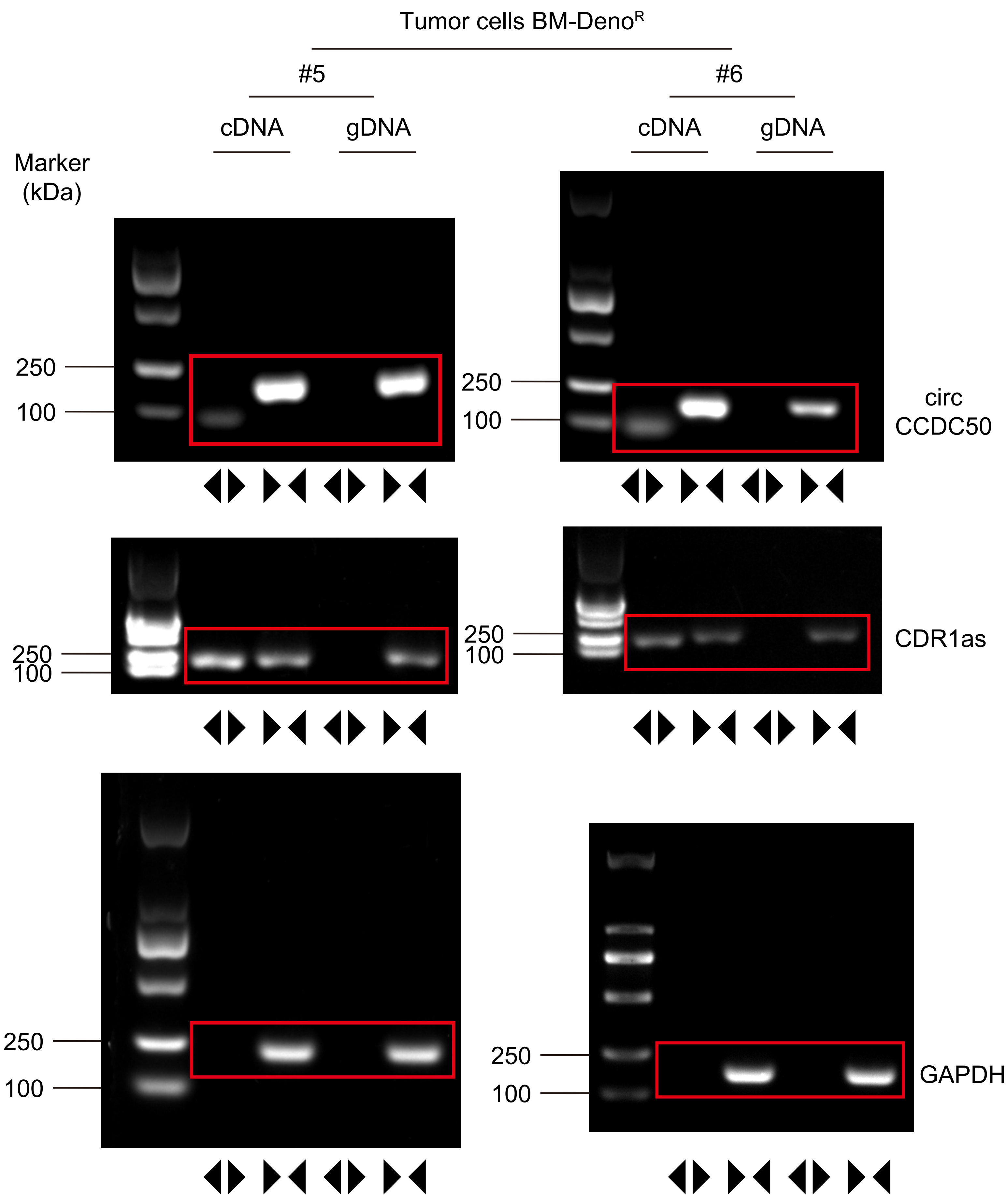

Full unedited blot for Supplementary Figure 6D

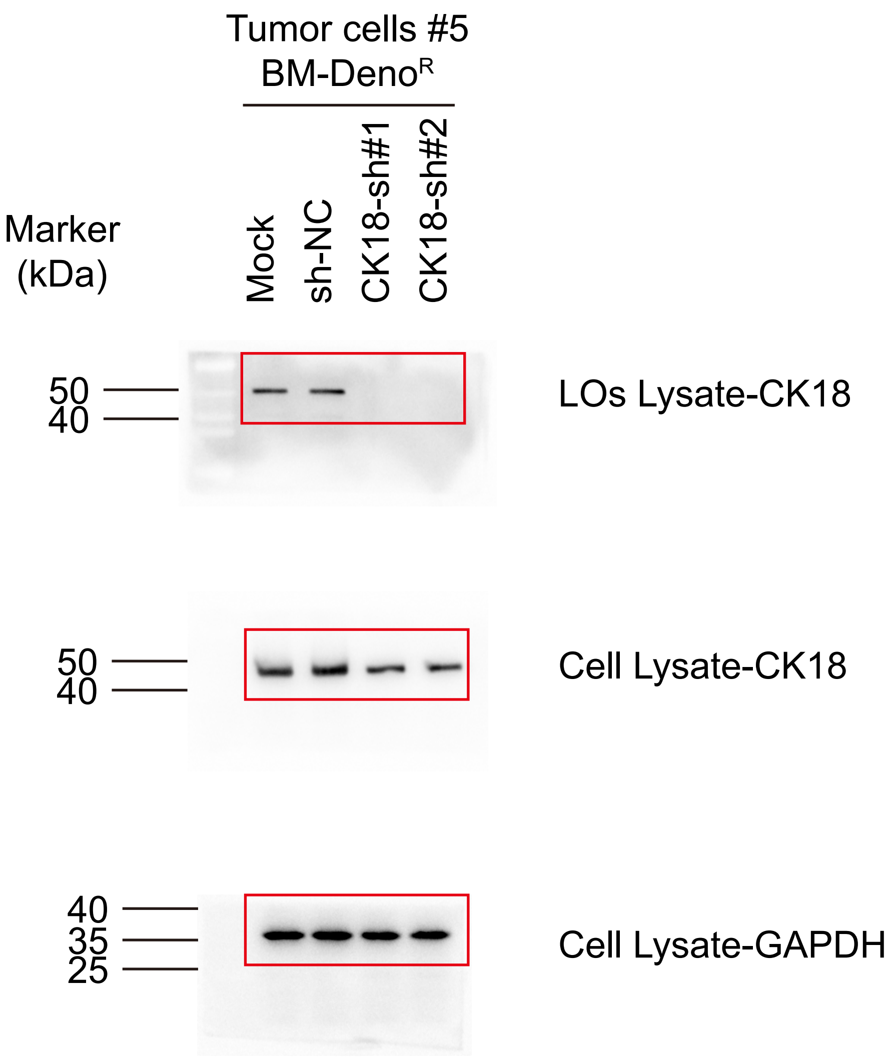

Full unedited blot for Supplementary Figure 6E

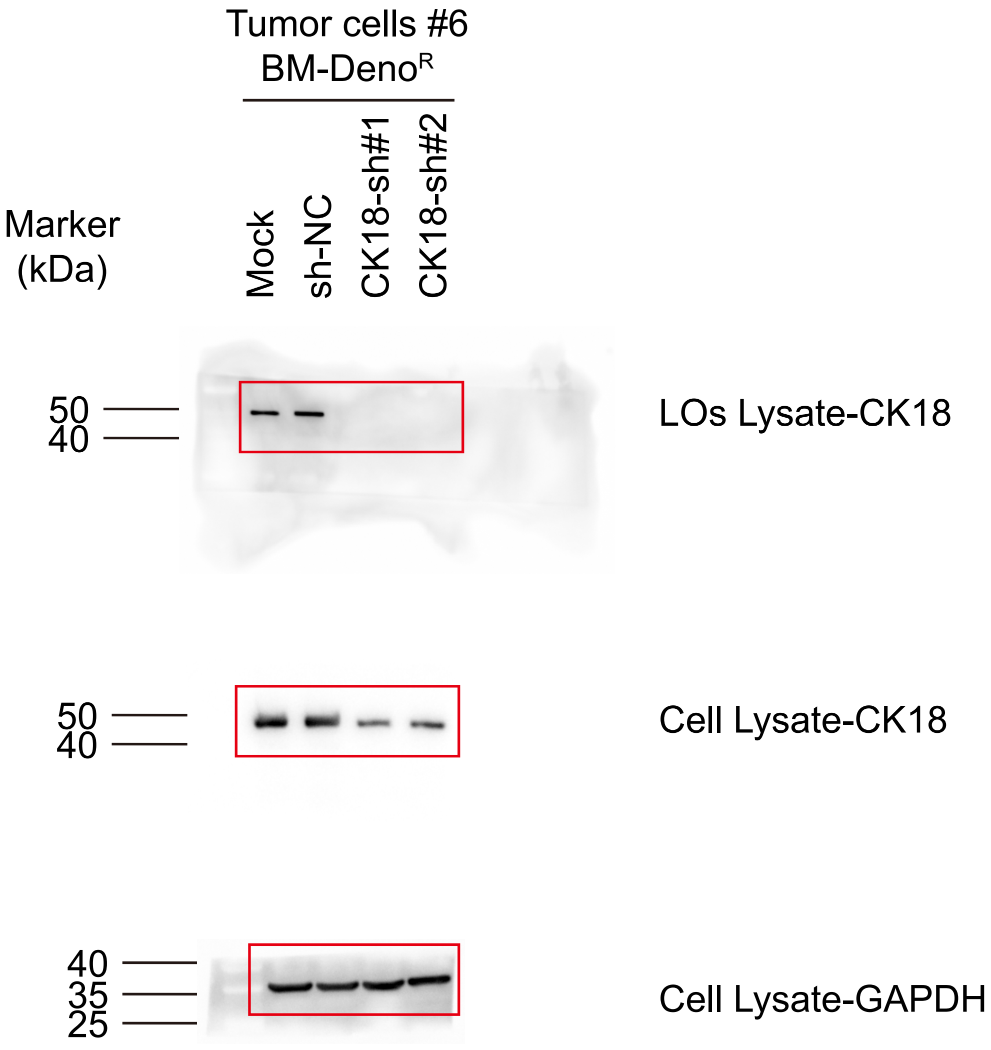

Full unedited blot for Supplementary Figure 8G

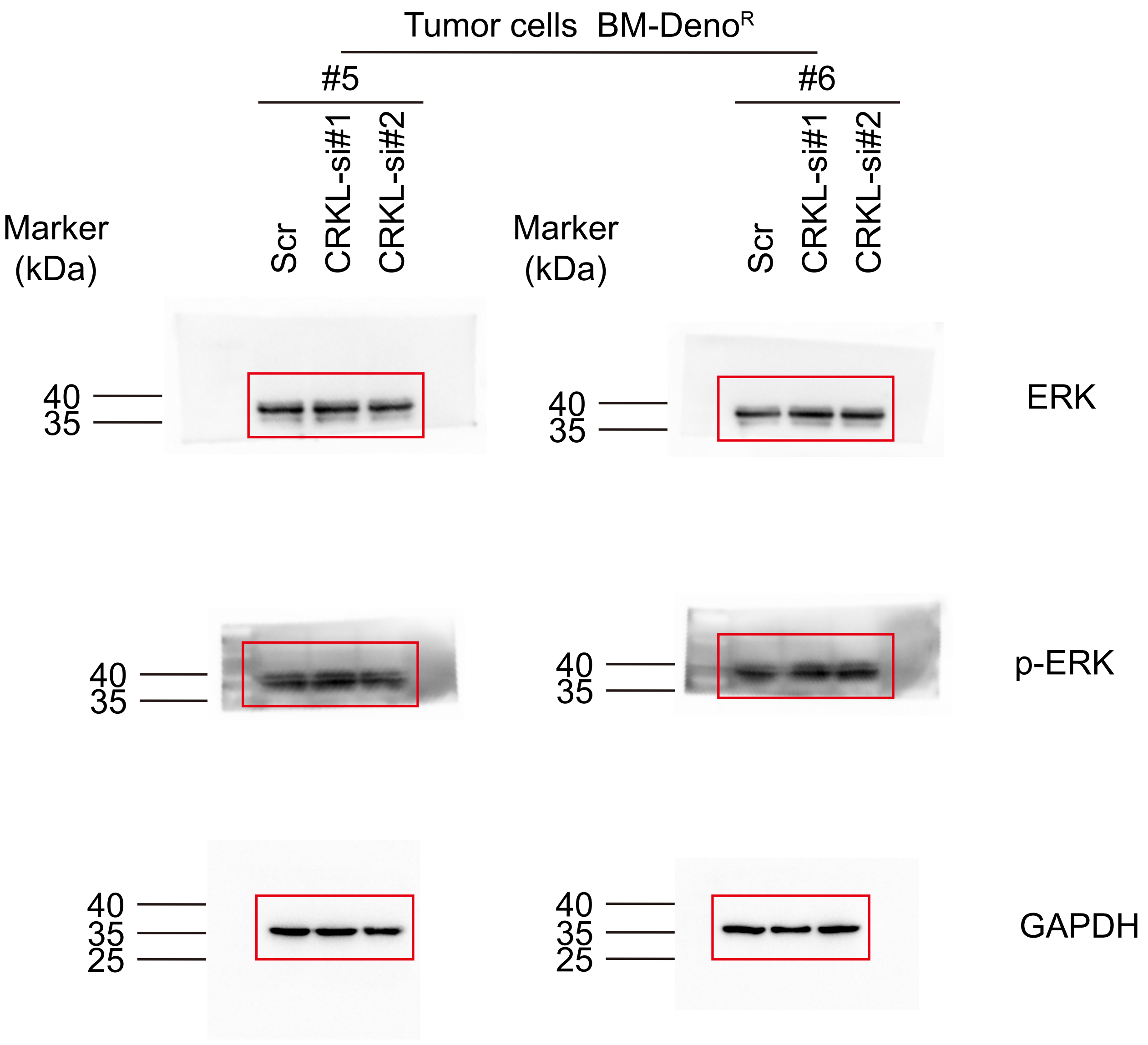

Full unedited blot for Supplementary Figure 8H

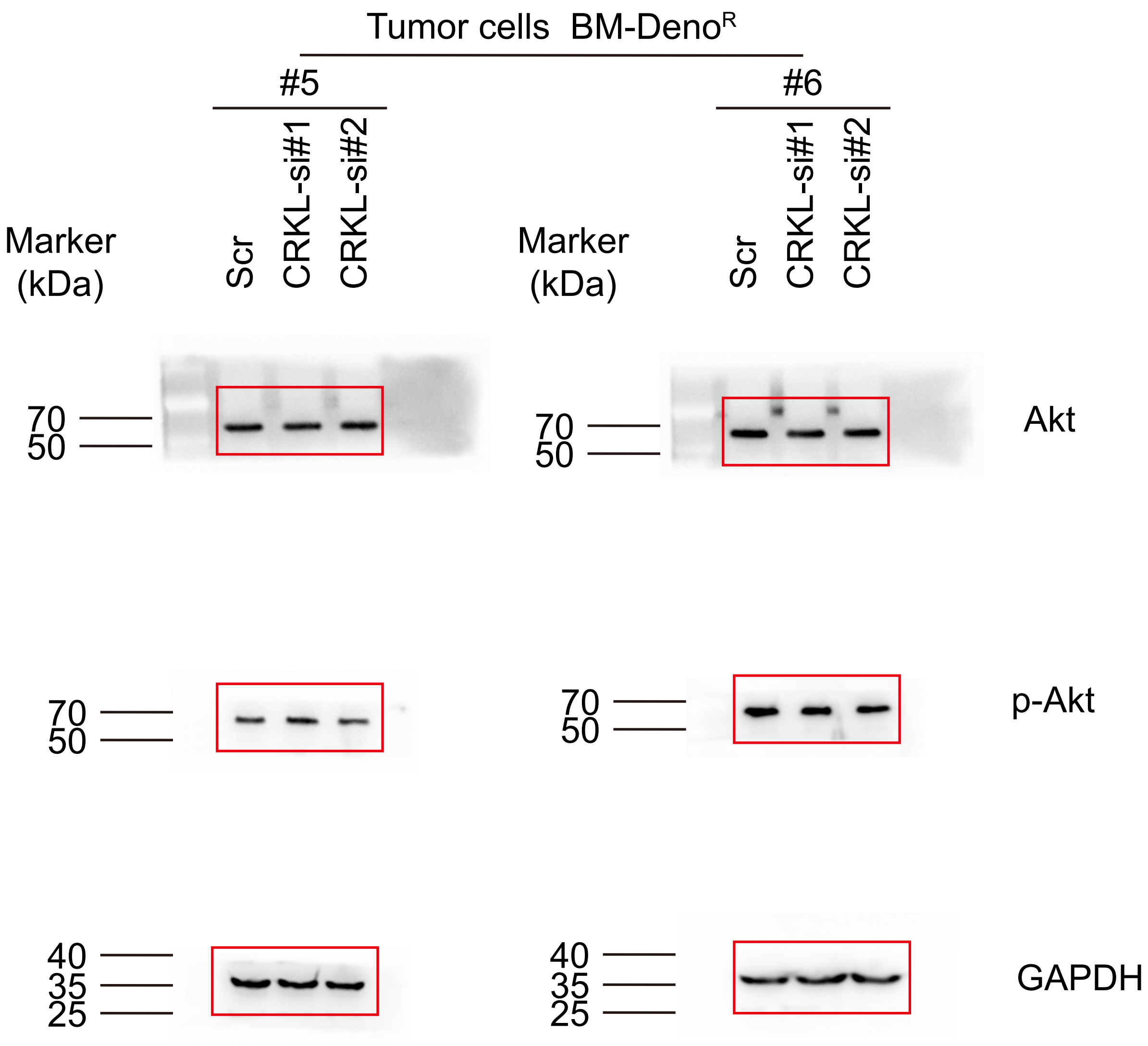

Full unedited blot for Supplementary Figure 10C

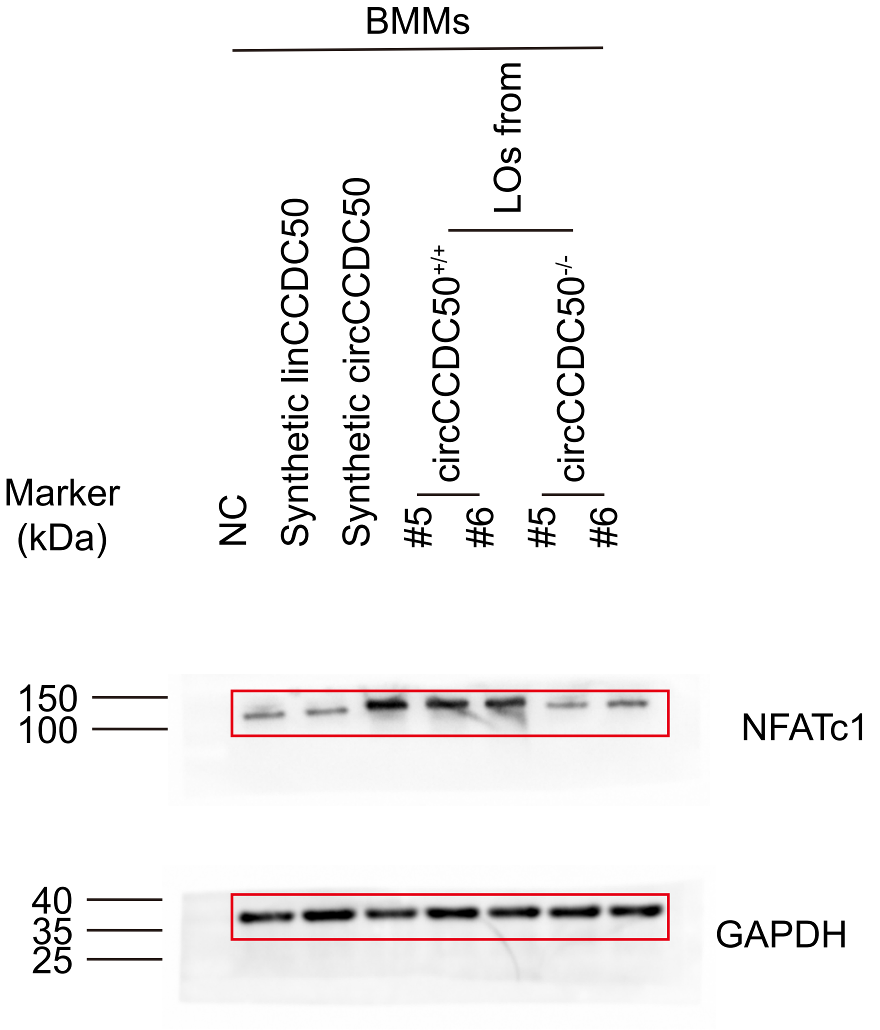

Full unedited blot for Supplementary Figure 10J

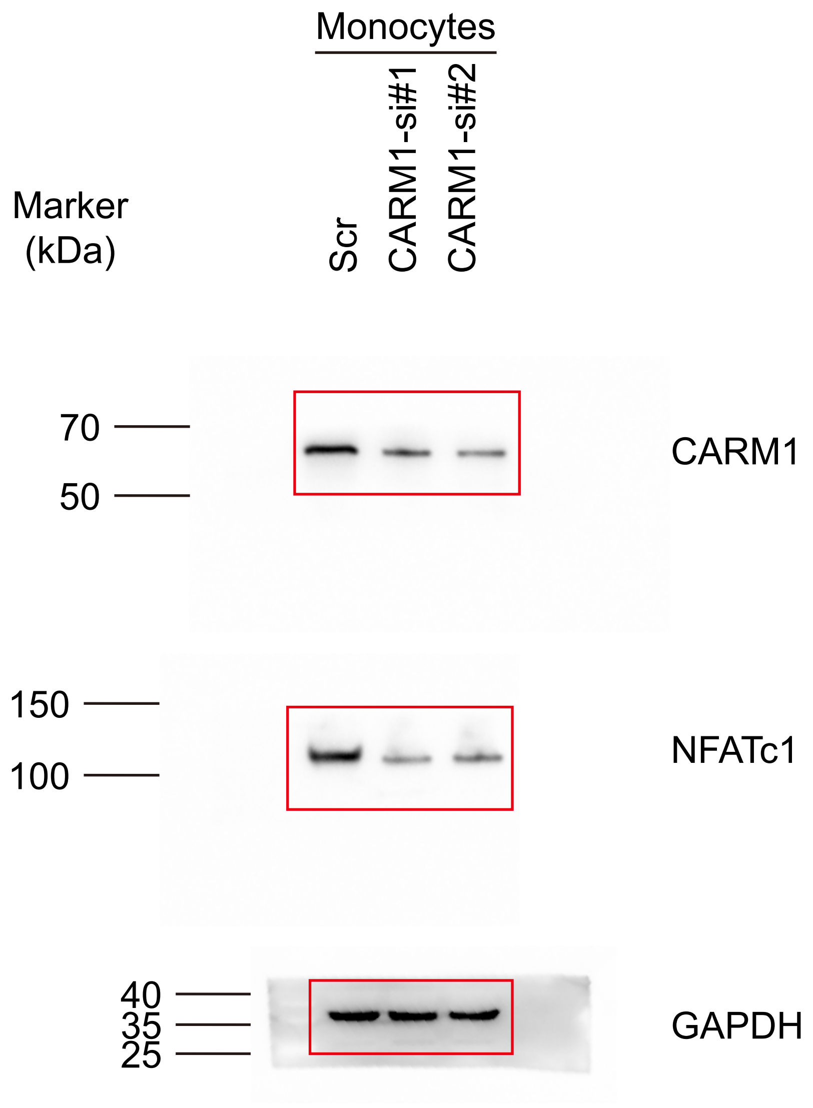

Full unedited blot for Supplementary Figure 10J

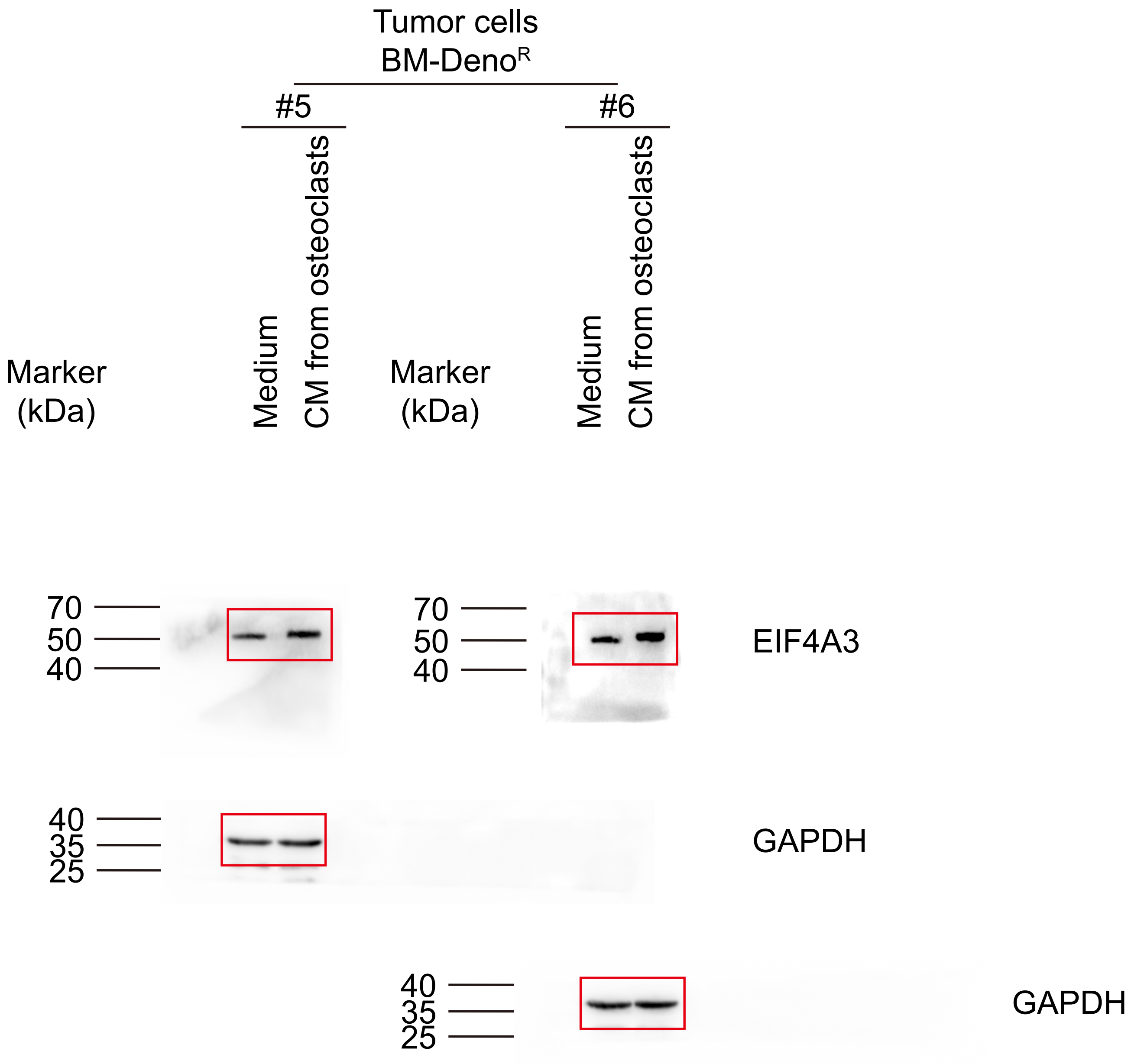

Full unedited blot for Supplementary Figure 10C

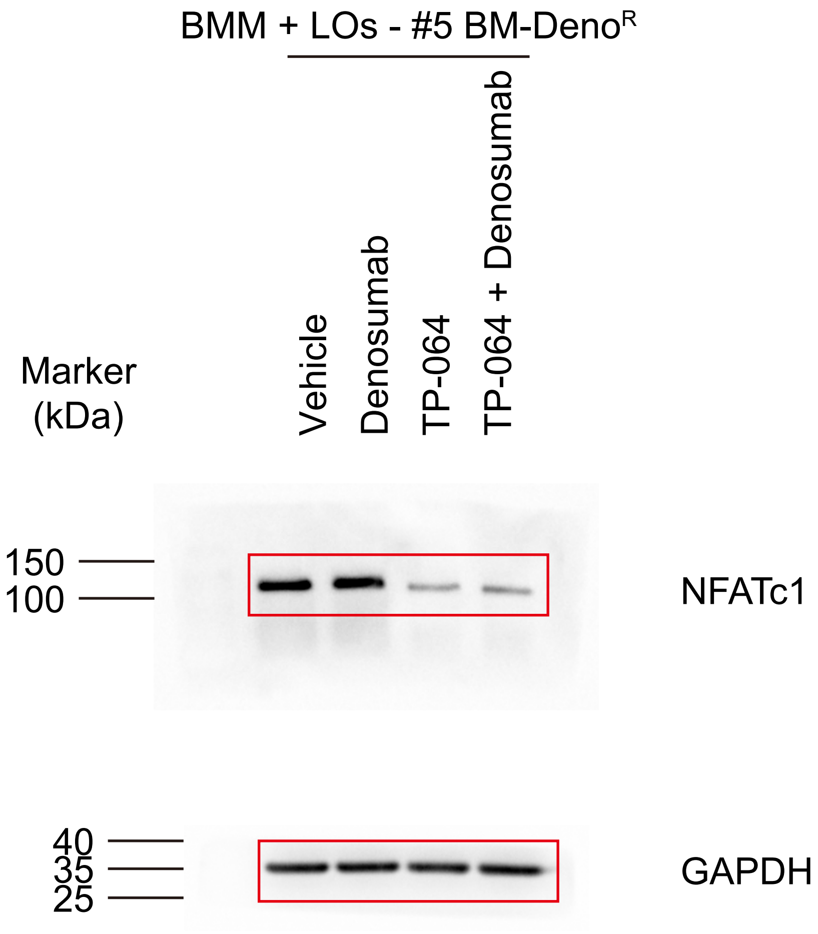

Full unedited blot for Supplementary Figure 10D

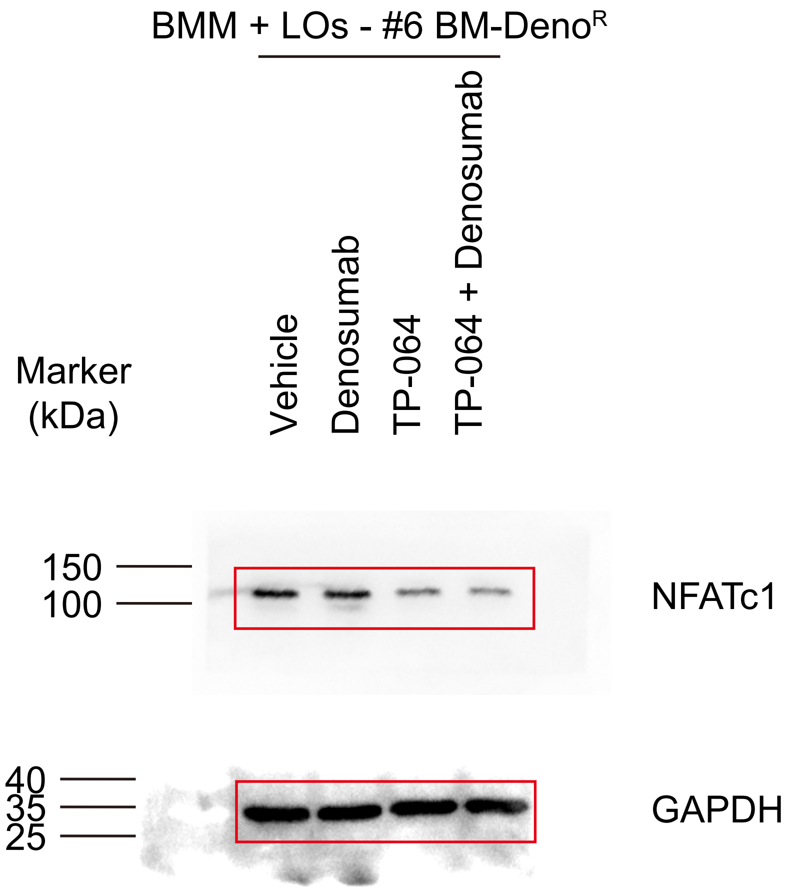

Supplement: Unedited blot and gel images [file jci-136-199285-s311.pdf]
